# Prolonged cardiac NR4A2 activation causes dilated cardiomyopathy in mice

**DOI:** 10.1007/s00395-022-00942-7

**Published:** 2022-07-01

**Authors:** Sadia Ashraf, Heinrich Taegtmeyer, Romain Harmancey

**Affiliations:** Division of Cardiology, Department of Internal Medicine, McGovern Medical School, The University of Texas Health Science Center at Houston, 6431 Fannin St., Houston, TX 77030 USA

**Keywords:** Dilated cardiomyopathy, Heart failure, Nuclear receptors, Cell cycle, Cardiac regeneration

## Abstract

Transcription factors play a fundamental role in cardiovascular adaptation to stress. Nuclear receptor subfamily 4 group A member 2 (NR4A2; NURR1) is an immediate-early gene and transcription factor with a versatile role throughout many organs. In the adult mammalian heart, and particularly in cardiac myocytes, NR4A2 is strongly up-regulated in response to beta-adrenergic stimulation. The physiologic implications of this increase remain unknown. In this study, we aimed to interrogate the consequences of cardiac NR4A2 up-regulation under normal conditions and in response to pressure overload. In mice, tamoxifen-dependent, cardiomyocyte-restricted overexpression of NR4A2 led to cardiomyocyte hypertrophy, left ventricular dilation, heart failure, and death within 40 days. Chronic NR4A2 induction also precipitated cardiac decompensation during transverse aortic constriction (TAC)-induced pressure overload. Mechanistically, NR4A2 caused adult cardiac myocytes to return to a fetal-like phenotype, with a switch to glycolytic metabolism and disassembly of sarcomeric structures. NR4A2 also re-activated cell cycle progression and stimulated DNA replication and karyokinesis but failed to induce cytokinesis, thereby promoting multinucleation of cardiac myocytes. Activation of cell cycle checkpoints led to induction of an apoptotic response which ultimately resulted in excessive loss of cardiac myocytes and impaired left ventricular contractile function. In summary, myocyte-specific overexpression of NR4A2 in the postnatal mammalian heart results in increased cell cycle re-entry and DNA replication but does not result in cardiac myocyte division. Our findings expose a novel function for the nuclear receptor as a critical regulator in the self-renewal of the cardiac myocyte and heart regeneration.

**Supplementary Information:**

The online version contains supplementary material available at 10.1007/s00395-022-00942-7.

## Introduction

In spite of multiple advances in the treatment of heart failure, prognosis remains poor with a 5-year mortality rate of 75% [47]. In the United States, where the adult heart failure population exceeds 6 million, deaths linked to heart failure have steadily increased over the past decade [48, 52]. This situation has stalled the decline in deaths from cardiovascular diseases and the increase in life expectancy [35, 48]. Dilated cardiomyopathy (DCM), one of the most common causes of heart failure, is defined by the presence of left ventricular (LV) or biventricular dilatation and systolic dysfunction in the absence of abnormal loading conditions or severe coronary artery disease. While a wide array of genetic and non-genetic factors have been implicated in the pathogenesis of DCM, most cases are still classified as idiopathic [12, 41].

The nuclear receptors of the NR4A subfamily NUR77 (NR4A1), NURR1 (NR4A2), and NOR1 (NR4A3) are encoded by immediate-early response genes and are involved in the regulation of a plethora of cellular processes. These receptors can function in a ligand-independent manner and their activity is regulated primarily through their expression levels, posttranslational modification events, and direct protein–protein interactions [26]. While acting principally via direct transcriptional activation or repression of target genes in the nucleus as monomers, homodimers or heterodimers with each other or the retinoid X receptor, NR4As also translocate to other cell compartments where they regulate protein stability and various biological processes such as autophagy, apoptosis and endoplasmic reticulum stress [39, 45]. In the mammalian heart, all three NR4A subfamily members are strongly up-regulated in response to beta-adrenergic stimulation, with cardiac myocytes representing a significant source of their expression [3, 33, 36]. The critical role of these nuclear receptors in defining cardiac adaptation or maladaptation to stress emerged only recently with the demonstration that NR4A1 protects the heart from isoproterenol-induced hypertrophy and contractile dysfunction [34, 57]. The fact that NR4A1 exacerbates, while NR4A3 protects from LV systolic dysfunction in ischemia also highlights the non-redundant functionality of these receptors in the heart [19, 61]. Interestingly, although cardiac NR4A2 was initially reported to be both the most rapidly and the most strongly activated of the NR4A members following beta-adrenergic stimulation [36], the physiologic consequences of this increase have remained largely unexplored.

Using isolated adult rat ventricular myocytes (ARVMs), we previously found that NR4A2 may act as potentially negative feedback regulator of beta-adrenergic mediated cell hypertrophy [3]. In the present study, we set out to confirm this finding in vivo. Contrary to our expectations, young adult male and female mice with time- and cardiac myocyte-specific overexpression of NR4A2 rapidly succumbed to death. Transverse aortic constriction (TAC) accelerated cardiac decompensation in hearts overexpressing NR4A2. Cardiac transcriptome and targeted cell signaling pathways analyses complemented by microscopic observations revealed that NR4A2 reversed adult cardiac myocyte metabolic and structural features to an immature phenotype. In addition, NR4A2 overexpression stimulated cell cycle re-entry resulting in increased DNA synthesis and cellular multinucleation. The cellular alterations were accompanied by apoptotic loss of cardiac myocytes and severe impairment of contractile function, which ultimately caused DCM and death from heart failure. Our findings point to NR4A2 as a master regulator of cardiac myocyte homeostasis. Its chronic activation in the adult heart has a detrimental impact on pump function and overall survival.

## Methods

An expanded methods section is available in the online Supplementary Information.

### Experimental animals

**Fig. 1:**
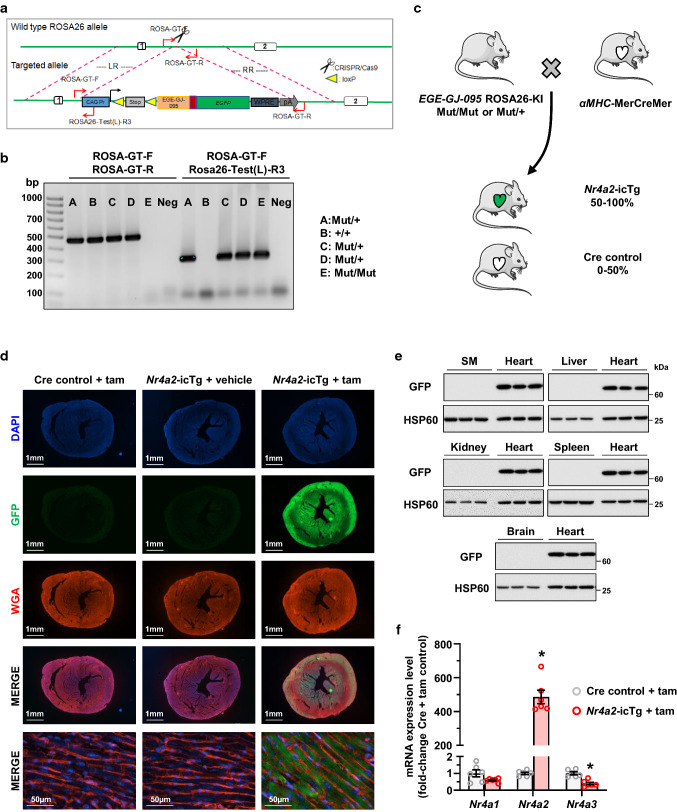
Validation of the tamoxifen-dependent, cardiac-restricted NR4A2 overexpression mouse model. **a** Schematic depicting the insertion of a bicistronic construct encoding the full-length murine NR4A2 cDNA sequence (*EGE-GJ-095*) and enhanced green fluorescent protein (GFP) reporter downstream of a CAG promoter and a floxed stop sequence at the Rosa26 locus. **b** Presence of the Rosa26 mutated allele (*Mut*) is confirmed by PCR using forward primer ROSA-GT-F (5′-AGTCGCTCTGAGTTGTTATCAG-3′) and reverse primer ROSA26-Test(L)-R3 (5′-GTCAATGGAAAGTCCCTATTGGCGT-3′) and subsequent visualization of a 278-base-pair (bp) amplicon on agarose gel. The non-mutated Rosa26 allele (+) is detected using the same forward primer paired with reverse primer ROSA-GT-R (5′-TGAGCATGTCTTTAATCTACCTCGATG-3′) and subsequent visualization of a 469 bp amplicon. **c** Schematic of breeding plan used to generate experimental animals used for the present study. **d** Representative images confirming expression of the transgene in all cardiac myocytes from *Nr4a2*-icTg mice after tamoxifen (tam) treatment. The nonuniform staining pattern of whole heart tissue sections is due to tissue autofluorescence. **e** Confirmation of the cardiac specificity of the transgene expression by Western blot quantification of GFP expression in *Nr4a2*-icTg mouse tissues following tam treatment. **f** Real-time PCR quantification of mRNAs encoding all 3 NR4A members in the left ventricle of mice at 21 days after tam treatment. Data are mean ± SEM of *n* = 6 animals per group and are expressed in fold change from expression levels detected in the LV of Cre recombinase expressing control mice. Data were analyzed by two-tailed Student *t* test. *P < 0.05 vs. Cre control + tam

All animal procedures were performed in compliance with the Guide for the Care and Use of Laboratory Animals and were approved by the Institutional Animal Care and Use Committees. The Nr4a2-reporter conditional knockin (*EGE-GJ-095* ROSA26-KI) mouse model was generated by Biocytogen (Wakefield, MA, USA) using the CRISPR/Cas9 based Extreme Genome Editing (EGE) technology. In brief, a bicistronic construct encoding the full-length murine NR4A2 and enhanced green fluorescent protein (GFP) reporter downstream of a CAG promoter and a floxed stop sequence was introduced at the Rosa26 locus (Fig. 1a). Presence of the Rosa26 mutated allele (*Mut*) was confirmed by PCR (Initial denaturation at 95 °C for 5 min, 30 cycles of denaturation-annealing-extension at 95 °C–62 °C–72 °C for 30 s each, and final extension at 72 °C for 10 min) using forward primer ROSA-GT-F (5′-AGTCGCTCTGAGTTGTTATCAG-3′) and reverse primer ROSA26-Test(L)-R3 (5′-GTCAATGGAAAGTCCCTATTGGCGT-3′) and subsequent visualization of a 278-base-pair (bp) amplicon on agarose gel. Presence of a non-mutated Rosa26 allele (+) was simultaneously tested for using the same forward primer paired with reverse primer ROSA-GT-R (5′-TGAGCATGTCTTTAATCTACCTCGATG-3′) and subsequent visualization of a 469 bp amplicon (Fig. 1b).

Cardiac myocyte targeted MerCreMer transgenic mice expressing tamoxifen-inducible Cre recombinase driven by the α-myosin heavy chain promoter were purchased from the Jackson Laboratory (Bar Harbor, ME, USA). The *αMHC*-MerCreMer mice were crossed with *EGE-GJ-095* ROSA26-KI animals (*Mut*/*Mut* and *Mut*/+) to generate mice with inducible cardiac-specific overexpression of NR4A2 (*Nr4a2*-icTg) and Cre expressing control animals (Fig. 1c). All animals were housed and bred on a 12-h light/12-h dark cycle at a temperature of 22 ± 2 °C and 40–60% humidity.

The expression of NR4A2 was induced in 8- to 9-week-old mice through a single intraperitoneal injection of tamoxifen (40 mg/kg body weight; MilliporeSigma, Burlington, MA, USA). Mice were randomized to the tamoxifen or vehicle (corn oil; MilliporeSigma) treatment using an Excel-generated spreadsheet. Tamoxifen-injected Cre control mice were also included to detect potential adverse cardiac effects induced by the Cre recombinase. Unless otherwise indicated, all cardiac functional and molecular analyses were carried out 3–4 weeks after NR4A2 transgene induction.

### Transthoracic echocardiography and Doppler imaging

Echocardiographic exams were performed under isoflurane anesthesia using a Vevo 3100 Imaging System (FUJIFILM VisualSonics, Toronto, Ont) according to the guidelines established by Lindsey et al. [30]. The amount of isoflurane dispensed (1–2% isoflurane in 100% O_2_) was individually adjusted to maintain similar heart rate between mice. Body temperature was kept within the physiologic range (36–37.5 °C) throughout the procedure using a dedicated heating pad. Pulsed wave and color flow Doppler imaging of the ascending and descending aorta were used after TAC surgery to determine blood pressure gradients across the constriction site. Successful TAC surgery was defined by a peak pressure gradient > 30 mmHg. B-Mode and M-Mode images obtained in the parasternal short axis (PSAX) view were used to determine LV anterior wall thickness at end-systole and end-diastole (LVAWs/d), LV internal diameter at end-systole and end-diastole (LVIDs/d), LV posterior wall thickness at end-systole and end-diastole (LVPWs/d), LV ejection fraction (LVEF), LV fractional shortening (LVFS), heart rate (bpm) and cardiac output (mL/min).

### RNA sequencing and analysis

Samples were pooled into single library using TruSeq Stranded mRNA Library Prep and sequenced with the NextSeq 500/550 Mid Output Kit v2.5 (150 cycles) on the Illumina NextSeq 500 platform (Illumina, San Diego, CA, USA). Sequenced reads were assessed for quality using the Illumina Basespace Cloud Computing Platform and FASTQ sequence files were used to align reads to the mouse reference genome [*Mus musculus*/UCSC mm9] using RNA-Seq Alignment Application with STAR aligner. Fragments per kilobase of transcript per million mapped reads (FPKM) values of reference genes and transcripts were generated using Cufflinks 2. Differential expression was determined by univariate analysis and a full list of differentially regulated genes (DRG; *P* < 0.01) is provided in Dataset S1. The generation of adult rat ventricular myocytes (ARVMs) overexpressing NR4A2 and RNA-Seq on those cells has previously been reported [3]. Molecular pathways differentially expressed between groups (*P* < 0.05) were identified and visualized using Reactome v76 (www.reactome.org).

### Antibody array

Left ventricular total protein and phosphorylation changes in 16 cell signaling pathways, including notably phosphoinositide 3-kinase (PI3K)/AKT signaling, apoptosis, autophagy, cell cycle, ErbB, focal adhesion, mitogen-activated protein kinase (MAPK), p53, and vascular endothelial growth factor (VEGF) signaling pathway were interrogated using the Cell Signaling Phospho Antibody Array from Full Moon Biosystems (Sunnyvale, CA; Array No. PCS300). Frozen tissue samples were shipped to Full Moon Biosystems for protein extraction and labeling, conjugation of biotin labeled proteins to the antibody array, detection using Cy3-streptavidin, array scanning and data acquisition. Raw signals and signals normalized to β-tubulin are provided in Dataset S2. Signals normalized to that of β-tubulin were uploaded into the BRB-ArrayTools v4.6.1 Excel plugin (National Institutes of Health, Bethesda, MD, USA) for class comparison analysis using the two-sample *t* test. *P* < 0.05 was considered statistically significant.

### Statistical analysis

All data are expressed as means ± SEMs and statistically analyzed with the use of GraphPad Prism software version 9 (GraphPad Software, San Diego, CA, USA). Comparisons between two groups were performed using a two-tailed Student *t* test. Comparisons between more than two groups were performed by one-way ANOVA followed by Tukey test. Serial comparisons of echocardiography data between more than two groups were carried out using two-way ANOVA followed by the Bonferroni test. *P* < 0.05 was considered significant.

## Results

### Validation of the tamoxifen-dependent, cardiac-restricted NR4A2 overexpression mouse model

Because all antibodies currently available to detect NR4A2 lack specificity, co-expression of a GFP reporter was used to track activation of the transgene in mouse tissues. As expected, GFP was exclusively expressed in the heart following treatment of *Nr4a2*-icTg mice with tamoxifen (Fig. 1d, e). Strong GFP signals were detected in all cardiac myocytes throughout the right and left ventricles (Fig. 1d). Three weeks after tamoxifen induction, NR4A2 mRNA expression levels increased 486-fold on average (Fig. 1f), which is about 5.5- to 7.5-fold higher than the activation of endogenous NR4A2 reported with isoproterenol treatment in cultured ARVMs and in mouse heart in vivo [3, 36]. Induction of NR4A2 did not affect cardiac NR4A1 expression but led to a 60% decrease in NR4A3 mRNA levels (Fig. 1f). Thus, the data confirmed successful time- and cardiac-specific induction of NR4A2 at near-physiological levels.

### Sustained NR4A2 activation leads to cardiac contractile dysfunction and death

**Fig. 2:**
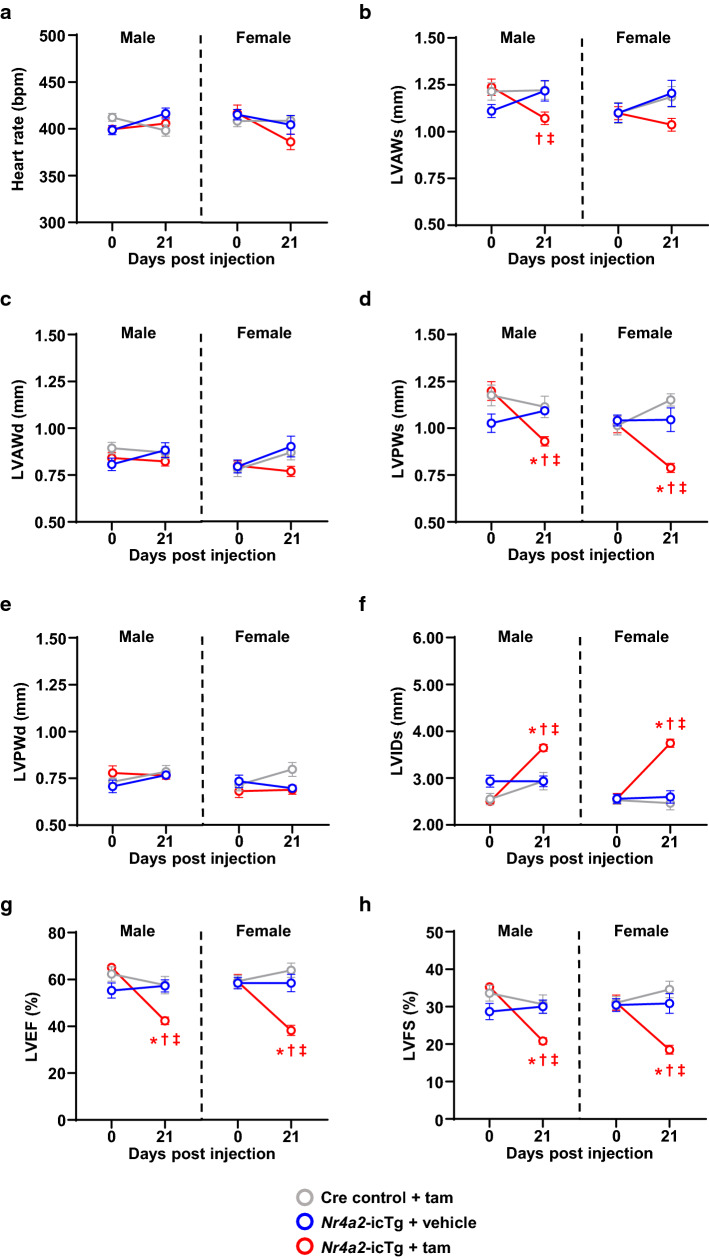
Cardiac myocyte-specific induction of NR4A2 in the adult heart leads to impaired left ventricular systolic function. Eight- to 9-week-old male and female *Nr4a2*-icTg mice underwent transthoracic echocardiography analysis of left ventricular (LV) function prior to (Day 0) and 21 days after transgene induction by tamoxifen (tam) injection. Left ventricular function of Cre recombinase expressing control mice and vehicle-treated *Nr4a2*-icTg mice was also recorded in parallel. Sex-specific changes in heart rate (**a**), LV anterior wall thickness at end-systole (LVAWs; **b**), LV anterior wall thickness at end-diastole (LVAWd; **c**), LV posterior wall thickness at end-systole (LVPWs; **d**), LV posterior wall thickness at end-diastole (LVPWd; **e**), LV internal diameter at end-systole (LVIDs; **f**), LV ejection fraction (LVEF; **g**), and LV fractional shortening (LVFS; **h**) are represented. Data are expressed as mean ± SEM of *n* = 10–12 mice per group. Data were analyzed by two-way repeated measures ANOVA with Bonferroni test. *P < 0.05 vs. Cre control + tam and ^†^*P* < 0.05 vs. *Nr4a2*-icTg + vehicle within same treatment day. ^‡^*P* < 0.05 vs. day 0 within same group

To gain insight into the impact of sustained NR4A2 activation for the adult heart, LV wall motion was evaluated serially by echocardiography before and after induction of the transgene. Mean heart rate values were similar between groups at each time point investigated (Fig. 2a). At 21 days after initiation of tamoxifen treatment, male and female *Nr4a2*-icTg mice displayed signs of contractile dysfunction, as evidenced by a decrease in LV walls thickening and greater LV internal diameter at end-systole, a 35% decrease in ejection fraction, and a ~ 40% decrease in fractional shortening (Fig. 2b–h).

**Fig. 3:**
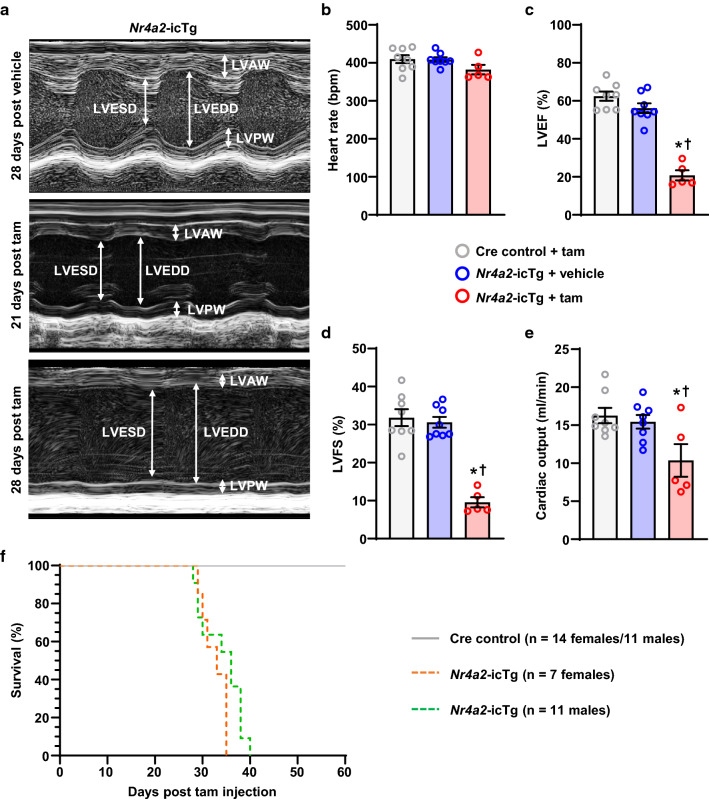
Cardiac myocyte-specific induction of NR4A2 in the adult heart leads to heart failure and death. **a** Representative M-mode images of parasternal short axis view at papillary muscle level in *Nr4a2*-icTg mice at 21 and 28 days after tamoxifen (tam) or vehicle injection. LVAW, left ventricular anterior wall; LVEDD, left ventricular diameter at end-diastole; LVESD, left ventricular diameter at end-systole; LVPW, left ventricular posterior wall. Comparison of heart rate (**b**), LV ejection fraction (LVEF; **c**), LV fractional shortening (LVFS; **d**), and cardiac output (**e**) between *Nr4a2*-icTg mice injected with tam (*n* = 2 males/3 females) or vehicle (*n* = 4 males/4 females) and Cre recombinase expressing control mice (*n* = 4 males/4 females) at 28 days following treatment. Data are expressed as mean ± SEM. Data were analyzed by one-way ANOVA with Tukey test. *P < 0.05 vs. Cre control + tam and ^†^*P* < 0.05 vs. *Nr4a2*-icTg + vehicle. **f** Kaplan–Meier curve comparing survival of male and female *Nr4a2*-icTg mice to that of Cre recombinase expressing control mice following tamoxifen injection

LV contractile function worsened quickly thereafter with extremely poor LV wall motion detected at 28 days post tamoxifen injection (Fig. 3a). At this point, LV ejection fraction and fractional shortening fell down to 20% and 10%, respectively, while mean cardiac output decreased by 33% (Fig. 3b–e). Death started to occur at 28 days after *Nr4a2* induction, with female mice dying on average 5 days earlier than their male counterparts. All *Nr4a2*-icTg mice were dead within 40 days (Fig. 3f).

### Sustained NR4A2 activation triggers dilated cardiomyopathy

**Fig. 4:**
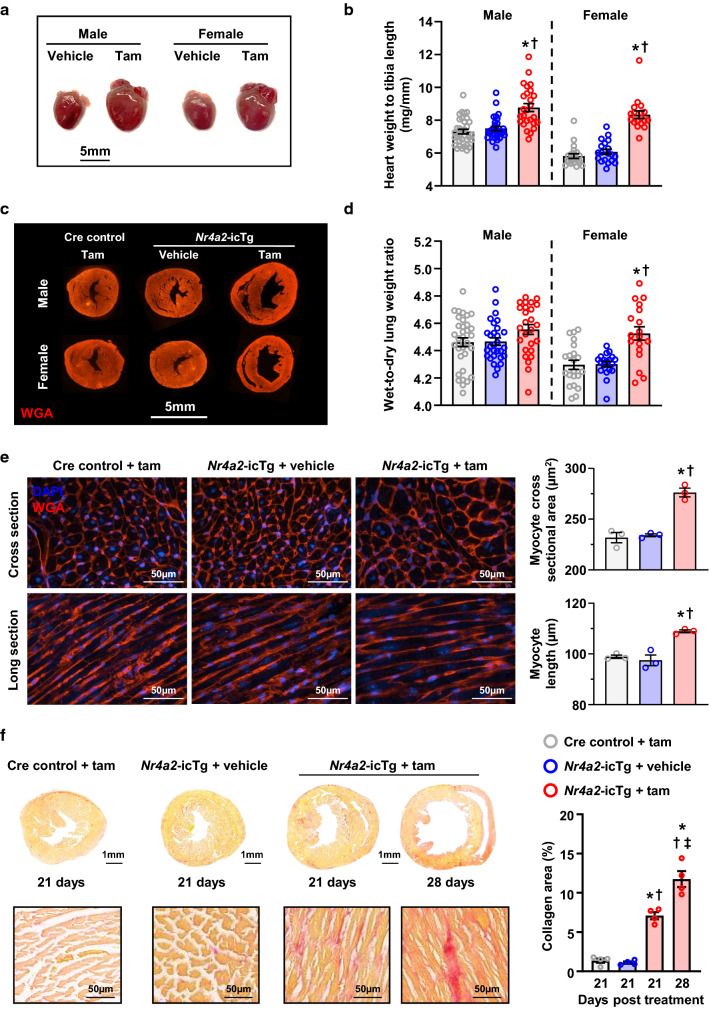
Cardiac myocyte-specific induction of NR4A2 in the adult heart causes structural remodeling consistent with development of dilated cardiomyopathy. **a** Representative images of hearts from tamoxifen (tam)- and vehicle-treated *Nr4a2*-icTg mice at 21 days following treatment. **b** Comparison of heart weight normalized to tibia length between *Nr4a2*-icTg mice injected with tam (*n* = 26 males/18 females) or vehicle (*n* = 29 males/19 females) and Cre recombinase expressing control mice (*n* = 34 males/21 females) at 21 days following treatment. **c** Representative cross-sectional images of wheat germ agglutinin (WGA)-stained hearts at 21 days following tam or vehicle treatment. **d** Comparison of wet-to-dry lung weight ratios between *Nr4a2*-icTg mice injected with tam (*n* = 26 males/18 females) or vehicle (*n* = 29 males/19 females) and Cre recombinase expressing control mice (*n* = 34 males/21 females) at 21 days following treatment. **e** Representative photomicrographs from hearts of male mice showing WGA- and DAPI-stained cardiac myocytes in cross-sectional and longitudinal orientations. Mean myocyte cross-sectional area and length were determined by averaging values from > 100 cells per animal (*n* = 3 animals per group). **f** Representative cross-sectional images and photomicrographs of Picrosirius red-stained hearts of male mice at 21 and 28 days after tamoxifen or vehicle treatment. Collagen quantification was performed on whole transverse cardiac sections from *n* = 4 animals per group. Data are expressed as mean ± SEM. Data were analyzed by one-way ANOVA with Tukey test. *P < 0.05 vs. Cre control + tam at 21 days, ^†^*P* < 0.05 vs. *Nr4a2*-icTg + vehicle at 21 days, and ^‡^*P* < 0.05 vs. *Nr4a2*-icTg + tam at 21 days

Gross and histopathological examinations of the heart were performed between the third and fourth week following NR4A2 induction. At 21 days, hearts from male and female mice appeared significantly enlarged (Fig. 4a). This enlargement was accompanied by increased heart weight after normalization either to body weight or to tibia length, all in absence of a significant change in body weight (Fig. 4b, Fig. S1a and Fig. S1b). Consistent with the rapid onset of heart failure, histopathology revealed a biventricular enlargement associated with thinning of the LV walls (Fig. 4c). In accordance with the survival analysis, the relative increase in cardiac weight was more pronounced among females and correlated with an increase in the wet-to-dry lung weight ratio, thus suggesting faster progression toward congestive heart failure in females than in males (Fig. 4b, d). At 28 days, increased heart weight in male and female mice was associated with a decrease in LV wall thickness and a concomitant increase in LV internal diameter and volume at end diastole (Fig. S2a–d). Wheat germ agglutinin (WGA) staining revealed an increase in both the length (+ 12%) and cross-sectional area (+ 18%) of cardiac myocytes associated with disruption of normal myocardial architecture (Fig. 4e). Picrosirius red (PSR) staining demonstrated this was accompanied by the appearance of interstitial fibrosis that was progressively complemented by replacement fibrosis between 21 and 28 days after induction of NR4A2 (Fig. 4f). Increased heart weight caused by an overall increase in fibrosis and myocyte hypertrophy with progressive wall thinning is indicative of the development of dilated cardiomyopathy.

To further investigate the pathophysiological relevance of these findings, cardiac NR4A2 mRNA levels were quantified in patients diagnosed with end-stage idiopathic dilated cardiomyopathy, both at time of implantation and explantation of a left ventricular assist device (LVAD). Mechanical unloading was accompanied by a trend to decreased NR4A2 expression in the left ventricle (Fig. S3).

### Sustained NR4A2 induction accelerates cardiac decompensation in pressure overload

**Fig. 5:**
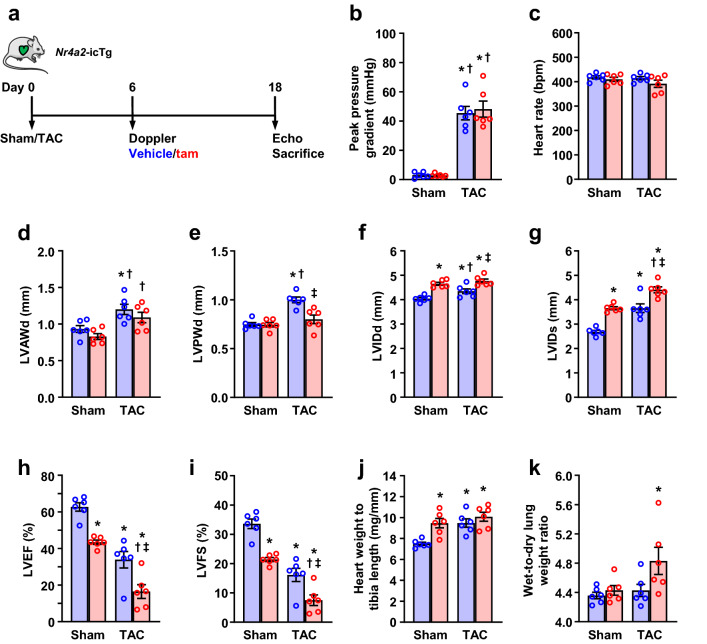
Chronic induction of NR4A2 in the adult heart accelerates cardiac decompensation during pressure overload. **a** Schematic of the experimental design. Eight- to 9-week-old male *Nr4a2*-icTg mice were subjected to transverse aortic constriction (TAC) or sham surgery at day 0. Successful induction of pressure overload was verified by Doppler analysis 6 days after surgery, at which point mice were randomly injected with tamoxifen (tam) or vehicle before undergoing transthoracic echocardiography and anthropometric analyses at day 18 post surgery. **b** Transaortic pressure gradients in TAC-operated *Nr4a2*-icTg mice compared to sham-operated *Nr4a2*-icTg mice. Echocardiographic determination of heart rate (**c**), left ventricular (LV) anterior wall thickness at end-diastole (LVAWd; **d**), LV posterior wall thickness at end-diastole (LVPWd; **e**), LV internal diameter at end-diastole (LVIDd; **f**), LV internal diameter at end-systole (LVIDs; **g**), LV ejection fraction (LVEF; **h**), and LV fractional shortening (LVFS; **i**) at end of the experiment. Comparison of heart weight normalized to tibia length (**j**) and wet-to-dry lung weight ratio (**k**) at end of the experiment. Data are expressed as mean ± SEM of *n* = 6 animals per group. Data were analyzed by one-way ANOVA with Tukey test. *P < 0.05 vs. sham-operated + vehicle, ^†^*P* < 0.05 vs. sham-operated + tam, and ^‡^*P* < 0.05 vs. TAC-operated + vehicle

Next, we evaluated whether sustained NR4A2 activation aggravates myocardial remodeling induced by pressure overload. To do so, male *Nr4a2*-icTg mice were subjected to TAC surgery 6 days prior to tamoxifen injection and myocardial structure and function were assessed 12 days after induction of the transgene (Fig. 5a). At the time of treatment, the mean pressure gradient was similarly elevated between *Nr4a2*-icTg mice that were randomly selected to receive tamoxifen or vehicle injection (Fig. 5b). At the end of the protocol, mean heart rate for all TAC-operated mice remained comparable to that of sham-operated *Nr4a2*-icTg animals (Fig. 5c). However, the TAC-mediated compensatory increase in LV anterior and posterior walls thickness, as present in vehicle-treated mice, was abrogated for mice with cardiac-specific NR4A2 overexpression (Fig. 5d–e). This was accompanied by a greater increase in LV internal diameter at end of diastole and end of systole, and by a further decrease in LV ejection fraction and fractional shortening (Fig. 5f–i). Although the increase in heart weight to tibia length ratio was not different between vehicle- and tamoxifen-treated, TAC-operated animals, the exacerbation of contractile dysfunction associated with chronic NR4A2 induction correlated with an increase in the wet-to-dry lung weight ratio (Fig. 5j, k). Therefore, sustained NR4A2 activity inhibited compensatory hypertrophy and accelerated cardiac maladaptation to pressure overload.

### Sustained NR4A2 activation reinstates an immature metabolic phenotype and leads to sarcomere disorganization in cardiomyocytes

**Fig. 6:**
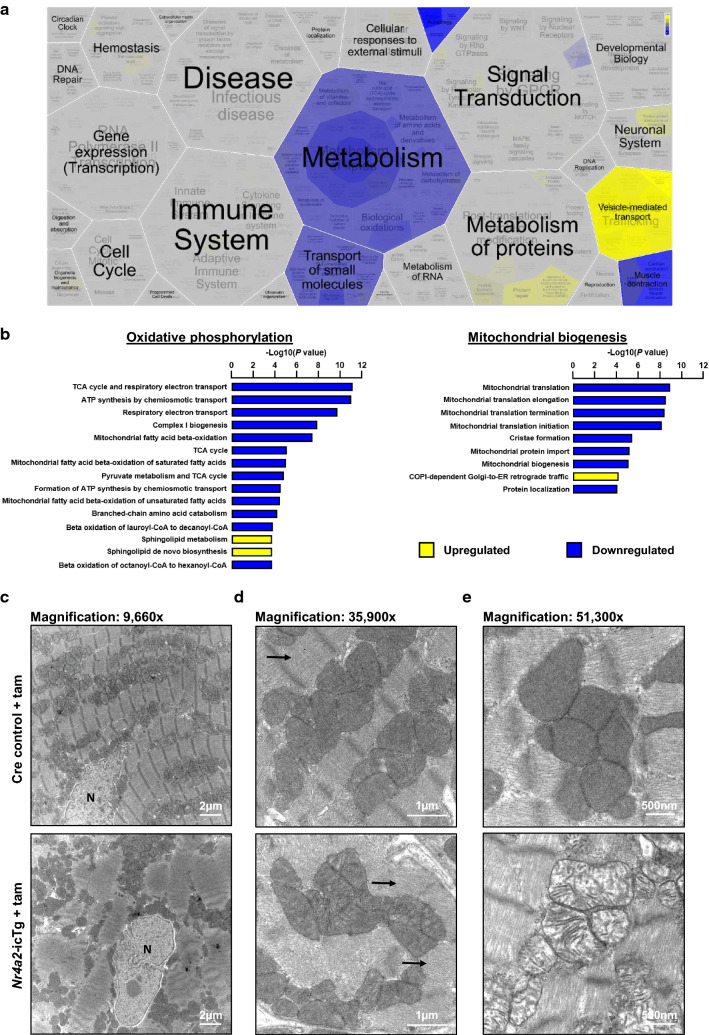
Chronic NR4A2 induction leads to metabolic and structural remodeling of adult cardiac myocytes in vivo. **a** Voronoi view of RNA sequencing data revealing the main biological processes differentially regulated in the left ventricle (LV) of male *Nr4a2*-icTg at 21 days after tamoxifen injection when compared to the LV of Cre recombinase expressing control mice. **b** Top Reactome pathways linked to oxidative phosphorylation and mitochondrial biogenesis that are significantly altered in the LV of *Nr4a2*-icTg at 21 days after tamoxifen injection when compared to the LV of Cre recombinase expressing control mice. Results generated from RNA sequencing analysis of *n* = 6 male mice per group. **c–e** Transmission electron microscopy visualization of subcellular structures in adult cardiac myocytes at 21 days after tamoxifen injection. **c** Cardiac myocytes in hearts of Cre recombinase expressing control mice show a tightly organized sarcomeric structure with clearly visible Z-lines, while cardiac myocytes in hearts of *Nr4a2*-icTg mice exhibit a more chaotic arrangement of myofibrils. **d** At higher magnification, myosin fibers are visible (arrows). However both longitudinal (upper arrow) and transverse (lower arrow) fibres are present within the same cardiac myocyte in hearts of *Nr4a2*-icTg mice which is indicative of disorganized sarcomeric structures. **e** At higher magnification the densely arrayed lamellar cristae of electron-dense control mitochondria are visible. However, mitochondria from *Nr4a2*-icTg mice display scarce lamellar cristae typical of immature organelles. N, cardiac myocyte nucleus

To gain more insight into the molecular changes associated with the rapid maladaptation of the NR4A2 overexpressing hearts, we analyzed the global transcriptomic signature of the LV at 21 days after transgene induction, i.e. at a time when both structural and functional alterations became apparent (Figs. 2, 4). Out of the 13,356 genes that passed filtering criteria for analysis, 6313 genes (47%) were found to be differentially expressed with a *p* value of ≤ 0.01 (Dataset S1). Main biological processes altered by NR4A2 induction included metabolism, muscle contraction, autophagy, the transport of small molecules (all down-regulated), and vesicle-mediated transport (up-regulated; Fig. 6a and Fig. S4a). Specifically, downregulation of oxidative phosphorylation, beta-oxidation of fatty acids, branched-chain amino acid catabolism and mitochondrial biogenesis were responsible for decreased metabolism (Fig. 6b). Decreased oxidative metabolism was compensated by a dramatic increase in anaerobic metabolism characterized by the concerted up-regulation of all but one glycolytic enzyme (Fig. S5 and Dataset S1). Although the expression of glucose transporters GLUT1 and GLUT4 was unchanged, translocation of GLUT4 to the plasma membrane was among the up-regulated vesicle-mediated transport processes (Fig. S4a, S4b). In accordance with the PSR staining, molecular pathways linked to collagen biosynthesis and extracellular matrix organization were also up-regulated (Fig. S4a). Real-time PCR quantification also confirmed activation of the cardiac fetal gene program (increased transcript amounts for *Nppa* and *Nppb* and decreased expression for *Atp2a2* and *Myh6*). Besides the loss of alpha-myosin heavy chain (*Myh6*), gene expression for several other contractile proteins abundantly expressed in the adult heart including cardiac troponin T (*Tnnt2*), cardiac troponin I (*Tnni3*) and tropomyosin-1 (*Tpm1*), was decreased (Fig. S4b).

To confirm our gene expression data, we assessed the ultrastructure of the cardiac myocytes by TEM. In comparison with hearts from Cre controls which displayed the well-organized microarchitecture typical of adult cardiac myocytes (with densely packed mitochondria between parallel-aligned myofibrils and regular t-tubules located at the level of the Z-lines), a large number of NR4A2-overexpressing myocytes exhibited chaotic arrangements of myofibrils and mitochondria with loss of well-defined sarcomeres and no recognizable t-tubular structures akin to idiopathic hypertrophic cardiomyopathy (Fig. 6c). As further evidence of disorganized sarcomeric structures, myosin fibers were simultaneously visible in both longitudinal and transverse orientation in the same myocyte (Fig. 6d). Lastly, the densely arrayed lamellar cristae of control mitochondria were replaced by scarce cristae typical of immature mitochondria in hearts of *Nr4a2*-icTg mice (Fig. 6e). Altogether, these findings suggest increased cellular plasticity and return to an immature phenotype for adult cardiac myocytes following chronic activation of NR4A2.

### Cardiac myocyte-specific induction of NR4A2 activates growth, proliferation and apoptosis signaling pathways

**Fig. 7:**
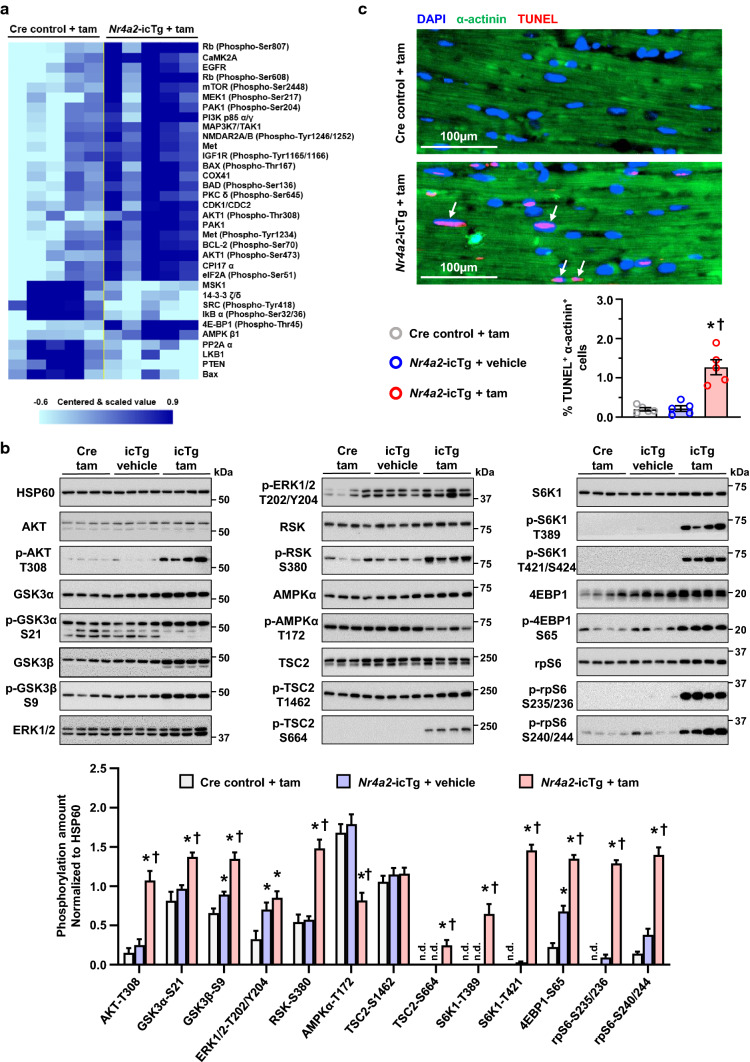
Cardiac myocyte-specific induction of NR4A2 activates growth, proliferation and apoptosis signaling pathways in the adult heart. **a** Heat map representation of the 34 proteins and phosphoproteins from 16 major cell signaling pathways that are differentially expressed in the left ventricle (LV) of *Nr4a2*-icTg mice when compared to LV of Cre recombinase expressing control mice at 21 days after tamoxifen (tam) treatment. Data from *n* = 5 male mice per group were normalized to individual β-tubulin signals and analyzed by a two-tailed Student *t* test. **b** Comparison of expression levels for proteins and phosphoproteins of the AKT, ERK1/2, AMPK, and mTOR related pathways in the LV of *Nr4a2*-icTg mice injected with tam or vehicle and Cre recombinase expressing control mice injected with tam at 21 days after treatment. Data are expressed as mean ± SEM of *n* = 8 animals per group. Data were analyzed by one-way ANOVA with Tukey test. *P < 0.05 vs. Cre control + tam and ^†^*P* < 0.05 vs. *Nr4a2*-icTg + vehicle. **c** Representative photomicrographs of sarcomeric α-actinin, DAPI- and TUNEL-stained LV tissue. Total number of TUNEL^+^ cardiomyocyte nuclei (white arrows) was determined by averaging values from > 1000 cardiomyocyte nuclei per animal (*n* = 5 animals per group). Data are expressed as mean ± SEM and were analyzed by one-way ANOVA with Tukey test. *P < 0.05 vs. Cre control + tam and ^†^*P* < 0.05 vs. *Nr4a2*-icTg + vehicle. *n.d.* not detectable

To further investigate possible intracellular signals responsible for the metabolic and structural remodeling of adult cardiac myocytes, we performed the unbiased quantification of 304 proteins and phosphoproteins from 16 major cell signaling pathways. Compared to Cre control animals, the expression and/or phosphorylation levels of 34 proteins was differentially affected with a *p* value of ≤ 0.05 in the LV of *Nr4a2*-icTg mice following tamoxifen treatment (Fig. 7a and Dataset S2). More specifically, increased phosphorylation of retinoblastoma protein (Rb) at serine residues 608 and 807 and increased expression of the cyclin-dependent kinase 1 (CDK1/CDC2) were overall indicative of increased progression through the G1 and G2/M checkpoints of the cell cycle, respectively. In addition, the activity of kinases known to be major positive regulators of cell growth and proliferation, including mitogen-activated protein kinase kinase 1 (MEK1), mechanistic target of rapamycin kinase (mTOR), phosphoinositide 3-kinase (PI3K), and the AKT kinase, was also increased (Fig. 7a). Increased activity of the AKT, extracellular signal-regulated kinase (ERK) and mTOR kinases and phosphorylation of their downstream targets were further confirmed by immunoblotting. In contrast, the antiproliferative AMP-activated protein kinase (AMPK) was inhibited (Fig. 7b).

While confirming the downregulation of genes linked to muscle contraction, Reactome analysis of our previously published transcriptomic data from ARVMs overexpressing NR4A2 also revealed global up-regulation of RNA metabolism [3]. Biosynthesis of ribosomal RNA (rRNA) and protein translation, both tightly coupled to cell growth and proliferation, represented the most activated molecular processes in those cells (Fig. S6).

In parallel to the uptick in growth and proliferation pathways, a decrease in BCL2-associated X, apoptosis regulator (BAX) expression, concomitant with an increased phosphorylation and regulation of BAX, BCL2-associated agonist of cell death (BAD) and BCL2 apoptosis regulator (BCL2), were also indicative of the activation of apoptosis and survival signals (Fig. 7a). Consistent with this last finding, the TUNEL assay revealed an ~ sixfold increase in apoptotic cardiomyocyte death in heart tissue from *Nr4a2*-icTg mice (Fig. 7c). In summary, cardiac myocyte-specific activation of NR4A2 was not only associated with stimulation of the cell cycle, increased cell growth and proliferation, but also increased apoptosis in the adult mouse heart.

### Sustained NR4A2 induction leads to multinucleation of adult cardiac myocytes

**Fig. 8:**
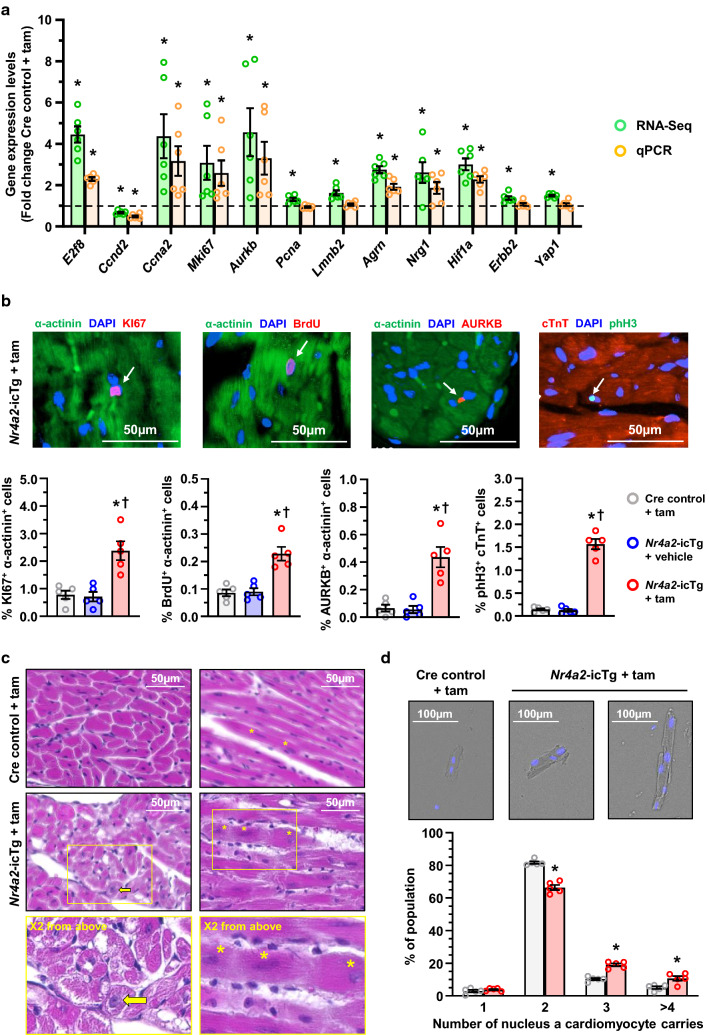
Chronic NR4A2 induction leads to cell cycle re-entry, polyploidization and multinucleation of adult cardiac myocytes. **a** Quantification by RNA sequencing and real-time PCR of mRNAs encoding cell cycle regulators (*E2f8*, *Ccnd2*, *Ccna2*), markers of cell cycle progression (*Mki67*, *Aurkb*, *Pcna*) and molecular enablers of proliferation (*Lmnb2*, *Agrn*, *Nrg1*, *Hif1α*, *Erbb2*, *Yap1*) in the left ventricle (LV) of *Nr4a2*-icTg mice at 21 days after tamoxifen (tam) treatment. Data are mean ± SEM of *n* = 6 animals per group and are expressed in fold change from expression levels detected in the LV of Cre recombinase expressing control mice. Data were analyzed by two-tailed Student *t* test. *P < 0.05 vs. Cre control + tam. **b** Representative photomicrographs of the immunohistochemical detection of Ki67 (KI67), Aurora kinase B (AURKB), BrdU and phosphorylated Histone H3 (phH3) in DAPI, sarcomeric α-actinin/cardiac troponin T (cTnT) stained LV tissue of *Nr4a2*-icTg mice at 21 days after tam treatment. Total numbers of cardiac myocytes nuclei^+^ (white arrows) were determined by averaging values from > 1000 nuclei per animal (*n* = 5 mice per group). Data are expressed as mean ± SEM and were analyzed by one-way ANOVA with Tukey test. *P < 0.05 vs. Cre control + tam and ^†^*P* < 0.05 vs. *Nr4a2*-icTg + vehicle. **c** Representative photomicrographs of hematoxylin and eosin-stained LV tissue at 21 days after tam treatment. Vacuolar degeneration caused by loss of myofibrils is visible in cardiac myocytes from *Nr4a2*-icTg mice. Yellow arrow points to an enlarged cardiac myocyte nucleus. Asterisks point to the nuclei of a tri-nucleated cardiac myocyte. **d** Representative bright-field photomicrographs of DAPI-stained cardiac myocytes isolated post-mortem from formalin-fixed hearts. Percentage of multinucleated cells was determined by averaging values from > 150 to 213 cardiac myocytes per animal (*n* = 5 animals per group)

Consistent with the protein data, targeted analysis of the cardiac transcriptome from *Nr4a2*-icTg mice revealed the differential regulation of several markers of adult cardiac myocytes proliferation following tamoxifen injection. Changes confirmed by real-time PCR quantification included the up-regulation of growth and proliferation signaling agents *Agrn*, *Nrg1*, and *Hif1a* (Fig. 8a). Cell cycle regulators encoded by *E2f8* and *Ccna2* were also increased, while *Ccnd2* was down-regulated. Expression of the genes encoding markers for proliferation (Ki67) and for midbody formation (Aurora kinase B; AURKB) were also increased (Fig. 8a). Fluorescence immunochemistry confirmed increased association of KI67 and AURKB with cardiac myocytes nuclei in *Nr4a2*-icTg mouse hearts, as well as increased nuclear incorporation of the phase S marker BrdU when compared to control animals (Fig. 8b).

Besides confirming the presence of vacuolar degeneration caused by a loss of myofibers in cardiac myocytes, H&E staining also revealed the presence of enlarged nuclei and multinucleation of cardiac myocytes in hearts of tamoxifen-treated *Nr4a2*-icTg mice (Fig. 8c). An increased number of cardiomyocyte nuclei stained for phosphorylated histone H3 was also detected in hearts of *Nr4a2*-icTg mice, thus further supporting an increased occurrence of acytokinetic mitosis in response to NR4A2 overexpression (Fig. 8b). Increased frequency of multinucleation events was confirmed by direct quantitation of DAPI^+^ nuclei in adult myocytes isolated at time of death from heart failure (Fig. 8d). Thus, chronic NR4A2 activation leads to increased DNA synthesis which is, in turn, accompanied by multinucleation of adult cardiomyocytes.

## Discussion

This study underscores the fundamental role of certain transcription factors in cardiovascular stress-response. We have shown that sustained expression of the nuclear receptor NR4A2 in the adult mouse heart leads to acute DCM and rapid death of the animals. The impairment of LV contractile function coincided with the reversal of cardiomyocytes to a fetal-like glycolytic metabolism and with the disorganization of sarcomeres. Chronic NR4A2 activation also induced widespread transcriptional alterations and caused terminally differentiated cardiomyocytes to re-enter the cell cycle even in the absence of cardiac stress. This resulted in enhanced karyokinesis but failed to induce cytokinesis, thereby promoting multinucleation of cardiac myocytes. Failure to progress through the cell cycle was accompanied by an increased number of cardiac myocytes undergoing apoptosis, which ultimately contributed to the etiology of DCM. There are several broad implications for these findings. First, they include a refined understanding of how environmental cues are integrated in the cardiac stress response. Second, they also include the potential for spatiotemporal modulation of NR4A2 activity as a way to stimulate heart regeneration.

While the roles of the NR4A nuclear receptors in the regulation of cardiac physiology are presently unknown, their functions in other organs and tissues, and particularly the liver, brain, skeletal muscle and the immune system have been fairly well established [17, 32]. As previously shown in those tissues, our findings demonstrate that cardiac NR4A2 simultaneously coordinates the regulation of a large number of diverse molecular pathways linked to metabolism, proliferation and apoptosis, all of which are known to play critical roles in the dedifferentiation, cell cycle re-entry, and ultimately determination of adult cardiac myocyte fate plasticity [14, 17]. Although metabolic and structural remodeling of adult cardiomyocytes is a typical feature of the mammalian heart under pathological conditions, evidence of increased cell cycle activity supports the notion that the observed cellular plasticity is linked to a genuine dedifferentiation process [64]. Our observations may have important implications for the development of cardiac regenerative strategies, because endogenous repair may occur primarily through dedifferentiation and proliferation of existing cardiac myocytes. Indeed, although some of the regulatory genes and signals needed to re-activate cardiac cell cycle progression have been identified, many laboratories are still actively probing for the fundamental molecular pathways that govern or suppress myocyte turnover [49]. Considering the known role of NR4A2 as an immediate-early response gene and the sheer extent of the cellular remodeling initiated by its activation, we postulate that NR4A2 acts as a master regulator of stress-induced cardiac myocyte self-renewal.

One of the most dramatic effects of NR4A2 overexpression consisted in the complete reshaping of cardiac metabolism, which reversed back from the highly oxidative capacity of the adult heart to a primarily glycolytic biosynthetic phenotype which is a feature of the fetal and failing heart. This finding is consistent with the established role of NR4A2 in the stimulation of glucose metabolism in skeletal muscle [2]. This switch in metabolic activity is critical for myocytes to re-enter the cell cycle, as recent studies performed in the adult zebrafish and postnatal mouse hearts demonstrated that inhibition of fatty acid oxidation and stimulation of glycolysis both promote proliferation of cardiomyocytes after injury [7, 13]. Loss of oxidative capacity coincided with the decrease in mitochondrial biogenesis and alteration of mitochondrial structure, which also reverted to a fetal-like appearance. This observation is also consistent with the known roles of mitochondrial biogenesis and expansion of cristae formation in reducing the proliferative capacity and driving the maturation of cardiac myocytes [27, 42, 43, 60]. Whether this metabolic remodeling is mediated by genomic regulation, non-genomic effects, or a combination of both remains to be determined. Indeed, direct stabilization of hypoxia-inducible factor-1α by NR4A2 may have contributed to the present phenotype [22].

In addition to muscle contraction being identified as another significantly down-regulated biological process, microscopic evidence confirmed the disorganization and loss of myofibrils in cardiac myocytes throughout the heart of tamoxifen-treated *Nr4a2*-icTg mice. Remodeling of the contractile apparatus is required for successful cell replication to occur, as stiffness of the myofibrils would otherwise impede nuclear division and cell cleavage. The majority of Z-bands have to undergo degradation during prometaphase, leading to the isolation and scattering of sarcomeres over subsequent phases of mitosis prior to their proper restoration in daughter cells [1, 50]. Disassembly and subsequent reorganization of the contractile machinery is under the control of signaling molecules diffusing through the extracellular matrix such as agrin (Agrn) and neuregulin 1 (Nrg1), both of which were up-regulated following NR4A2 activation [5, 46]. The disassembly and detachment of sarcomeric structures can be clearly observed during regeneration of the Zebrafish heart following ventricular resection [21]. However, while dedifferentiation and proliferation of adult myocytes may have little impact on cardiac function when concentrated at the site of injury, such mechanism may result in loss of contractile function when occurring at the whole-heart scale [49]. Thus, widespread dedifferentiation of myocytes resulting in decreased cardiac bioenergetics and a loss of functional rigor likely contributed to the impairment of systolic function and development of heart failure in *Nr4a2*-icTg mice.

In spite of the presence of metabolic and structural signatures typical of proliferating cells, enhanced cell cycle activity, and DNA replication in the heart, none of these markers represent a direct surrogate for new myocytes formation, and even increased detection of AURKB is not always indicative of successful cytokinesis [11, 18]. Unlike during the embryonic and neonatal stages, energetic reliance on glycolysis and activation of proliferation pathways including the ERK1/2, PI3K/AKT and mTOR pathways in the postnatally stressed heart are usually associated with hypertrophic rather than hyperplasic growth [9, 25, 31]. This results in increased polyploidization and multinucleation of myocytes in the injured myocardial tissue, two nuclear events which are paradoxically known to contribute to the loss of proliferative capacity of those cells [16, 28, 53]. Although polyploidization and multinucleation may promote cardiac adaptation to stress by making cardiomyocytes more resistant to injury [20, 28], this was clearly not the case for *Nr4a2*-icTg mice as increased NR4A2 activation accelerated cardiac decompensation during chronic pressure overload.

Several factors may have contributed to incomplete cell cycle progression in the present mouse Tg model. First, hypoxia-inducible factor (HIF)-1 α has a divergent role in cardiac proliferation and development, and its activation can promote cell cycle arrest through Myc antagonism [15, 24]. Second, transcript levels for the cell cycle activator Cyclin D2 (*Ccnd2*), a protein critical for persisting cardiac myocytes cell cycle activity, were decreased [63]. Lastly, expression of the atypical E2 factor 8 (*E2f8*), a cell cycle regulator essential for polyploidization in mammalian cells, was increased [38]. Cell cycle regulation and programmed cell death share many signal transduction mechanisms [51], and we also found evidence of increased activation of both pro-survival and pro-apoptotic cellular pathways resulting in increased myocytes apoptosis in hearts of *Nr4a2*-icTg mice. Cardiac myocyte regeneration and death are so intimately linked that both processes are believed to occur simultaneously [37]. Although the exact series of molecular events that increased apoptosis remains to be determined, the diffuse loss of cardiac myocytes likely played a critical role in the development of DCM and heart failure [37, 54, 56]. It is also unclear whether the concomitant downregulation of NR4A3 plays a role in the disease mechanism since activation of this particular NR4A member has been linked to the protection of cardiomyocytes from cellular stress and death [6].

It is noteworthy that female mice appeared to be more susceptible to the chronic effects of cardiac NR4A2 as they displayed more severe symptoms of heart failure at 21 days after transgene induction, and died 12.5% faster than male mice. There is some evidence that the regulation of the *Nurr1* gene is controlled by gender-specific hormones, such as estrogen and testosterone, and that these regulatory mechanisms may account for phenotypical differences between male and female [40, 44]. Whether a sex difference exist in the regulation of cardiovascular physiology by endogenous NR4A2 remains to be determined.

Few studies have reported myocyte cell cycle re-entry as a direct contributor to heart failure in vivo. Similar to our results, murine cardiac myocytes lacking GSK-3 were also reported to undergo karyokinesis but had impaired mitotic capacity and failed to progress to cytokinesis, which resulted in mitotic catastrophe, widespread apoptosis, and rapid development of DCM [62]. In humans, DCM caused by excessive DNA replication in cardiac myocytes, a phenomenon that has been termed mitogenic cardiomyopathy, has been rarely reported since such defects cause death in the early infancy [8, 59]. The expression of Myc, another early response gene activated as the adult heart remodels, also leads to cell cycle re-entry and increased DNA replication when induced in post-mitotic murine cardiac myocytes. Interestingly, Myc re-activation has been associated either with adaptive hypertrophy, with atrophy, or with hypertrophic cardiomyopathy and heart failure in different reports [10, 29, 55]. This last finding highlights the importance of other factors such as timing and level of induction of cell cycle regulators in determination of the cardiac myocyte fate. The chronic high NR4A2 expression in the heart of *Nr4a2*-icTg mice is a critical parameter which most likely had a significant impact on the outcome of this study. Indeed, we previously reported that a both shorter and more physiological induction of NR4A2 inhibited ERK1/2 hypertrophic signaling in ARVMs, which is in contrast with the increased ERK1/2 activity observed in the present model [3]. Mechanical unloading of the failing human heart, a process associated with normalization of cardiomyocyte function and size, also tended to decrease NR4A2 expression [4, 58]. Based on these observations, we postulate that a more transient and moderated activation aimed at recapitulating the stimulation of NR4A2 by the adrenergic system as reported by Myers and colleagues, possibly by using one of the known small-molecule activators of NR4A2, may increase survival of myocytes and promote adaptation of the stressed heart [23, 36].

In summary, we report that myocyte-specific overexpression of NR4A2 in the postnatal mammalian heart results in increased cell cycle re-entry and DNA replication but does not result in cardiac myocyte division. Chronic NR4A2 activation leads to activation of cell cycle checkpoints and induction of an apoptotic response resulting in loss of cardiac myocytes, impairment of cardiac function and heart failure. While additional studies will be essential to determine the impact of physiological “bouts” of NR4A2 induction for cardiac adaptation to injury, our findings highlight a novel function for the nuclear receptor as a critical regulator in the self-renewal of the cardiac myocyte and heart regeneration.

## Supplementary Information

Below is the link to the electronic supplementary material.

Supplementary file1 (PDF 1177 KB)

Supplementary file2 (XLSX 541 KB)

Supplementary file3 (XLSX 119 KB)

## Abbreviations

AMPK: AMP-activated protein kinase
ARVM: Adult rat ventricular myocyte
AURKB: Aurora kinase B
BAD: BCL2-associated agonist of cell death
BAX: BCL2-associated X, apoptosis regulator
BCL2: BCL2 apoptosis regulator
BrdU: Bromodeoxyuridine
CDK1/CDC2: Cyclin-dependent kinase 1
DAPI: 4′,6-Diamidino-2-phenylindole
DCM: Dilated cardiomyopathy
ERK: Extracellular signal-regulated kinase
FITC: Fluorescein isothiocyanate
GFP: Green fluorescent protein
H&E: Hematoxylin and eosin
LV: Left ventricle
LVAWs/d: Left ventricular anterior wall thickness at end-systole and end-diastole
LVAD: Left ventricular assist device
LVEF: Left ventricular ejection fraction
LVFS: Left ventricular fractional shortening
LVIDs/d: Left ventricular internal diameter at end-systole and end-diastole
LVPWs/d: Left ventricular posterior wall thickness at end-systole and end-diastole
MEK1: Mitogen-activated protein kinase kinase 1
mTOR: Mechanistic target of rapamycin kinase
NR4A1, 2, 3: Nuclear receptor subfamily 4 group A members 1, 2, 3
PI3K: Phosphoinositide 3-kinase
PSR: Picrosirius
Rb: Retinoblastoma protein
TAC: Transverse aortic constriction
TEM: Transmission electron microscopy
TUNEL: Terminal deoxynucleotidyl transferase dUTP nick end labeling
WGA: Wheat germ agglutinin
