# Supplementary material for: Prolonged cardiac NR4A2 activation causes dilated cardiomyopathy in mice
Source: Basic Res Cardiol. 2022 Jul 1;117(1):33. doi: 10.1007/s00395-022-00942-7 (PMC9249728; doi:10.1007/s00395-022-00942-7)
Supplement: Supplementary file 1 — Supplementary file1 (PDF 1177 KB) [file 395_2022_942_MOESM1_ESM.pdf]

Prolonged Cardiac NR4A2 Activation Causes Dilated Cardiomyopathy in Mice

Sadia Ashraf, Heinrich Taegtmeyer, Romain Harmancey

Department of Internal Medicine, Division of Cardiology, McGovern Medical School at The University of Texas Health Science Center at Houston, Houston, TX, USA.

Running title: Cardiac NR4A2 overexpression causes cardiomyopathy

Corresponding Author:
Dr. Romain Harmancey
Department of Internal Medicine, Division of Cardiology
McGovern Medical School at UTHealth,
6431 Fannin St., Houston TX 77030, USA
(Phone) +1713-500-6409
E-mail: romain.harmancey@uth.tmc.edu
ORCID: 0000-0002-5040-4500

Supplementary Methods

Survival analysis

Starting at 21 days after induction of the transgene, mice were monitored every day for heart failure symptoms. Mice with suspected heart failure showed lower levels of spontaneous locomotor activity, dyspnea, and rough fur. For ethical considerations and in accordance with the Institutional Animal Care and Use Committee study protocol, death from heart failure was not used as an endpoint. Instead, mice were treated every 10-12 hours with buprenorphine (0.1 mg/kg) starting at onset of symptoms, and isolated in a cage with softer bedding and moist food. Sick mice were then monitored every 10-12 hours for progression of symptoms until they became moribund, at which point the animals were euthanized. Time at euthanasia was considered time of death from heart failure.

Transverse aortic banding surgery.

Pressure overload was induced in 8- to 9-week-old male mice by transverse aortic constriction (TAC). In brief, mice were anesthetized with 2-3% isoflurane and injected subcutaneously with sustained-release buprenorphine (1.0-1.2 mg/kg) as analgesic prior to beginning of surgery. After shaving and disinfection of the skin surrounding the surgical site, a midline anterior cervical incision was performed to reveal the trachea and facilitate placement of a tracheal tube, connecting the mouse to a ventilator. The incision was extended caudally and a median sternotomy initiated to reveal the thymus. Retractors were placed to separate the thymus from the heart and reveal the aortic arch. A 7-0 silk suture was placed around the aorta between the first and the second branch, and tied firmly around a 27-gauge needle. The latter was promptly removed to yield a constriction of 0.4 mm in diameter. Sham-operated mice underwent a similar surgical procedure without constriction of the aorta. The chest and neck were closed with a 5-0 silk suture, and mice were allowed to recover from anesthesia before returning to their cages. During the whole procedure and recovery period, the mice were placed on a 38°C heating pad to maintain body temperature constant.

In vivo BrdU incorporation.

Two weeks after tamoxifen or vehicle treatment, mice were intraperitoneally injected with 100 µl of a 10 mg/ml bromodeoxyuridine (BrdU) solution diluted in 1X DPBS (BD Biosciences, Franklin Lakes, NJ, USA) for 3 consecutive days. Mice were euthanized one day after the last BrdU injection and tissue samples processed for immunohistochemistry as indicated below.

Histology and immunohistochemistry.

Tissues were excised from euthanized mice, washed in ice-cold saline and fixed with 10% neutral buffered formalin at room temperature (RT) for 48 h, embedded in paraffin and sectioned at 5 µm. Picro-sirius red staining was performed using kit catalog No. ab245887 from abcam (Cambridge, MA, USA) according to the manufacturer's instructions. For immunohistochemistry, sections were put to heat-induced antigen retrieval and blocking with 1% BSA. The sections were incubated with specific primary antibodies overnight at 4°C. After washing with PBS, the sections were incubated with secondary antibodies and/or fluorescein isothiocyanate (FITC) labelled wheat germ agglutinin lectin (Vector Laboratories, Burlingame, CA, USA) for 1 h at RT, washed and sealed with a mounting medium containing DAPI (Vector Laboratories). Terminal deoxynucleotidyl transferase dUTP nick end labeling (TUNEL) assay was performed using a click chemistry kit

(Catalog no. C10618) according to the manufacturer's instructions (Invitrogen, Waltham, MA, USA). Sections were imaged on a Lionheart FX Automated Microscope and analyzed with Gen5 data collection and analysis software (BioTek Instruments, Winooski, VT, USA). Detailed information about antibodies and reagents used for immunohistochemistry is provided in Table S1.

RNA isolation and purification.
Mouse hearts were excised from euthanized mice, the LV dissected and immediately flash-frozen in liquid nitrogen. Total RNA was extracted from frozen LV tissue with a Bio-Gen PRO200 Homogenizer (PRO Scientific, Oxford, CT, USA), DNase I-treated and purified using Qiagen reagents and spin columns (Qiagen, Germantown, MD, USA). Total RNA integrity and concentration were determined on a Qubit fluorometer (ThermoFisher Scientific, Waltham, MA, USA).

Transmission electron microscopy.
Samples for transmission electron microscopy (TEM) imaging were fixed by immersion in a solution of 2% glutaraldehyde, 2% paraformaldehyde in a 0.1M Sodium Cacodylate buffer (pH 7.2) for a minimum of 24 h at 4°C. Samples were then washed three times with Cacodylate buffer to clear excess fixative. During processing, samples were post-fixed in a 1% aqueous solution of osmium tetroxide for 1 hour and washed again with three buffer exchanges. Subsequently, samples were dehydrated in a graded ethanol series (50, 70, 90, 95, 100%) and propylene oxide was used as a transition solvent between the ethanol and Embed 812 resin. Samples were allowed to sit overnight in a 50:50 propylene oxide:resin solution until all the propylene oxide had evaporated. They were then incubated in fresh resin for 2 hours at room temperature before final embedding. Polymerization took place at 65°C for 24 hours. Thin sections (100nm) made with Leica UC6 Ultracut ultramicrotome (Leica Microsystems, Wetzlar, Germany) were placed on 200 mesh copper grids, post stained with 2% Uranyl Acetate followed by Reynolds Lead Citrate, and examined on a *Tecnai G^2 Spirit* TWIN *(FEI Company, Hillsboro, OR, USA) operating at an accelerating voltage of 80kV*. Images were acquired digitally with an AMT digital imaging system.

Real-time PCR.
One microgram of total RNA was reverse-transcribed with Superscript IV reverse transcriptase using random hexamers as per manufacturer's instructions (ThermoFisher Scientific). Predesigned TaqMan gene expression assays (Table S2) were employed for relative quantification of mRNAs encoding selected target genes with the standard curve method on a StepOne Real-Time PCR System (ThermoFisher Scientific). For mouse samples, gene expression levels were normalized through quantification of Peptidylprolyl Isomerase A (Cyclophilin A; *Ppia*) as the housekeeping gene. For human samples, gene expression levels were normalized using the geometric mean of housekeeping genes encoding Cyclophilin A, Tyrosine 3-Monooxygenase/Tryptophan 5-Monooxygenase Activation Protein Zeta (*Ywhaz*), and Glyceraldehyde-3-Phosphate Dehydrogenase (*Gapdh*).

Western blotting.
Frozen LV tissue samples were homogenized with a Bio-Gen PRO200 Homogenizer in protein lysis buffer containing 2.5 mmol/L EGTA, 2.5 mmol/L EDTA, 20 mmol/L KCl, 40 mmol/L beta-glycerophosphate, 40 mmol/L NaF, 4 mmol/L NaPPi, 10% (v/v) glycerol, 0.1% (v/v) Nonidet-

P40, cOmplete protease inhibitor cocktail and phosphatase inhibitor cocktails 2 and 3 (MilliporeSigma). Tissue homogenates were centrifuged at 16,000 x g for 5 min at 4ºC and protein concentration of the supernatant determined by bicinchoninic acid assay (Thermo Fisher Scientific). Proteins were separated by polyacrylamide gel electrophoresis in reducing sample buffer and transferred to 0.45 µm pore size polyvinylidene difluoride membranes. Membranes were blocked for 1 h with 5% (w/v) milk in 1X Tris-buffered saline, 0.4% (v/v) Tween 20 at RT and incubated overnight at 4ºC with primary antibodies diluted in blocking solution. Protein detection was carried out on the following day using horseradish peroxidase-conjugated secondary antibodies (Cell Signaling Technology, Danvers, MA, USA) and chemiluminescent substrate (Thermo Fisher Scientific). Densitometry band quantifications were performed with ImageJ v1.53g (National Institutes of Health). All signals were normalized to that of heat shock protein 60 (HSP60). Detailed information about antibodies used for Western blotting is provided in Table S1.

Cardiac myocyte isolation and counting of nuclei.

Formalin-fixed ventricular tissue was thoroughly minced with surgical scissors and cells further separated in 1X PBS by gentle homogenization with a glass pestle tissue grinder. The tissue lysate was filtered with 1X PBS on a 100 µm nylon mesh cell strainer and the filtrate subsequently centrifuged at 1000g for 5 min. Cells were resuspended in 1X PBS and the nuclei were stained with DAPI prior to visualization for counting on a Lionheart FX Automated Microscope.

Human heart tissue samples.

Cardiac tissue samples were obtained from 5 patients with idiopathic dilated cardiomyopathy referred to the Texas Heart Institute for heart transplantation and placed on left ventricular assist device (LVAD) support for a mean duration of 236±171 days (see Table S3 for individual demographic and clinical data). Tissue from the left ventricular apex was obtained during LVAD implantation and again at LVAD explantation. Tissue samples were immediately frozen in liquid nitrogen and stored at -80°C prior to total RNA extraction following same protocol as for mouse heart tissue. The protocol was approved by the Committee for the Protection of Human Subjects of St. Luke's Episcopal Hospital in Houston, Texas, and of The McGovern Medical School at UTHealth.

Table S1 List of antibodies and reagents used in Western blot (WB) and fluorescence immunohistochemistry (FIHC) analyses

Target Protein	Antibody Full Commercial Name	Vendor	Catalog No.	RRID	Application	Dilution/ Concentration Used
AKT	Akt1/2/3 (H-136)	SC	sc-8312	AB_671714	WB	1:1000
Phospho-AKT (T308)	Phospho-Akt (Thr308) (244F9)	CST	#4056	AB_331163	WB	1:1000
GSK3α	GSK-3α (D80E6)	CST	#4337	AB_10859910	WB	1:1000
Phospho-GSK3α (S21)	Phospho-GSK-3α (Ser21) (D1G2)	CST	#8452	AB_10860247	WB	1:1000
GSK3β	GSK-3β (D5C5Z) XP	CST	#12456	AB_2636978	WB	1:1000
Phospho-GSK3β (S9)	Phospho-GSK-3β (Ser9) (D85E12) XP	CST	#5558	AB_10013750	WB	1:1000
ERK1/2	P44/42 MAPK (Erk1/2) (137F5)	CST	#4695	AB_390779	WB	1:1000
Phospho-ERK1/2 (T202/Y204)	Phospho-p44/42 MAPK (Erk1/2) (Thr202/Tyr204) (D13.14.4E) XP	CST	#4370	AB_10234795	WB	1:1000
TSC2	Tuberin/TSC2 (D93F12) XP	CST	#4308	AB_10547134	WB	1:1000
Phospho-TSC2 (T1462)	Phospho-Tuberin/TSC2 (Thr1462) (5B12)	CST	#3617	AB_490956	WB	1:1000

Phospho-TSC2 (S664)	Recombinant Anti-Tuberin (phosphor S664) [EPR8202]	Abcam	Ab133465	AB_11157389	WB	1:1000
RSK	RSK1/RSK2/RSK3 (32D7)	CST	#9355	AB_659900	WB	1:1000
Phospho-RSK (S380)	Phospho-p90RSK (Ser380) (D5D8)	CST	#12032	AB_2797804	WB	1:1000
S6K1	p70 S6 Kinase (49D7)	CST	#2708	AB_390722	WB	1:1000
Phospho-S6K1 (T389)	Phospho-p70 S6 Kinase (Thr389) (108D2)	CST	#9234	AB_2269803	WB	1:1000
Phospho-S6K1 (T421/S424)	Phospho-p70 S6 Kinase (Thr421/Ser424)	CST	#9204	AB_2265913	WB	1:1000
rpS6	S6 Ribosomal Protein (5G10)	CST	#2217	AB_331355	WB	1:1000
Phospho-rpS6 (S240/244)	Phospho-S6 Ribosomal Protein (Ser240/244) (D68F8) XP	CST	#5364	AB_10695727	WB	1:1000
Phospho-rpS6 (S235/236)	Phospho-S6 Ribosomal Protein (Ser235/236) (D57.2.2E) XP	CST	#4858	AB_916156	WB	1:1000
AMPKα	AMPKα (D5A2)	CST	#5831	AB_10622186	WB	1:1000
Phospho-AMPKα (T172)	Phospho-AMPKα (Thr172) (40H9)	CST	#2535	N/A	WB	1:1000

HSP60	HSP60 (D6F1) XP	CST	#12165	AB_2636980	WB	1:1000
4E-BP1	4E-BP1 (53H11)	CST	#9644	AB_2097841	WB	1:1000
Phospho-4E-BP1 (S65)	Phospho-4E-BP1 (Ser65)	CST	#9451	N/A	WB	1:1000
GFP	GFP (D5.1)	CST	#2956	AB_1196615	WB/FIHC	1:1000/1:200
AURKB	Anti-Aurora B	abcam	ab2254	AB_302923	FIHC	1:200
Ki67	Recombinant Anti-Ki67 [SP6]	abcam	ab16667	AB_302459	FIHC	1:200
BrdU	Anti-BrdU [IIB5]	abcam	ab8152	AB_308713	FIHC	1:10
phH3	Alexa Fluor 488 Anti-Histone H3 (phospho S10 + T11)	abcam	ab200614-488	N/A	FIHC	1:5000
α-actinin	Sarcomeric Alpha Actinin [EA-53]	abcam	ab9465	AB_307264	FIHC	10 µg/ml
cTnT	Cardiac Troponin T	abcam	ab45932	AB_956386	FIHC	1 µg/ml
Anti-rabbit IgG, DyLight 488 labeled	Horse Anti-Rabbit IgG (H+L), DyLight 488	Vector Laboratories	DI-1088	AB_2336403	FIHC	1:250
Anti-rabbit IgG, DyLight 594 labeled	Horse Anti-Rabbit IgG (H+L), DyLight 594	Vector Laboratories	DI-1094	AB_2336414	FIHC	1:250

Anti-mouse IgG, DyLight 594 labeled	Horse Anti-Mouse IgG (H+L), DyLight 594	Vector Laboratories	DI-2594	AB_2336412	FIHC	1:250
WGA	Fluorescein wheat germ agglutinin	Vector Laboratories	FL-1021	AB_2336866	FIHC	10 µg/ml
Mounting medium with DAPI	Vectashield Antifade Mounting Medium with DAPI	Vector Laboratories	H-1200	AB_2336790	FIHC	N/A

SC, Santa Cruz Biotechnology; CST, Cell Signaling Technology; N/A, Not Applicable/Not Available; RRID, Research Resource Identifier.

Table S2 List of predesigned TaqMan Gene Expression Assays used for real-time PCR quantification of murine gene transcript levels

Gene Symbol	Species	Gene Name	Manufacturer Assay ID*
Nr4a1	Mouse	nuclear receptor subfamily 4, group A, member 1	Mm01300401_m1
Nr4a2	Mouse	nuclear receptor subfamily 4, group A, member 2	Mm00443060_m1
Nr4a2	Human	nuclear receptor subfamily 4, group A, member 2	Hs01117527_g1
Nr4a3	Mouse	nuclear receptor subfamily 4, group A, member 3	Mm00450071_g1
E2f8	Mouse	E2F transcription factor 8	Mm01204160_m1
Ccnd2	Mouse	cyclin D2	Mm00438070_m1
Ccna2	Mouse	cyclin A2	Mm00438063_m1
Mki67	Mouse	antigen identified by monoclonal antibody Ki 67	Mm01278617_m1
Aurkb	Mouse	aurora kinase B	Mm01718146_g1
Pcna	Mouse	proliferating cell nuclear antigen	Mm00448100_g1
Lmnb2	Mouse	lamin B2	Mm00456766_m1
Agrn	Mouse	Agrin	Mm01264855_m1
Nrg1	Mouse	Neuregulin 1	Mm01212130_m1
Hif1a	Mouse	hypoxia inducible factor 1, alpha subunit	Mm00468869_m1
Erbb2	Mouse	v-erb-b2 erythroblastic leukemia viral oncogene homolog 2	Mm00658541_m1
Yap1	Mouse	yes-associated protein 1	Mm01143263_m1
Ppia	Mouse	peptidylpropyl isomerase A (Cyclophilin A)	Mm02342430_g1
Ppia	Human	Peptidylpropyl isomerase A	Hs01565700_g1
Slc2a1	Mouse	Solute carrier family 2 member 1, GLUT1	Mm00441480_m1
Slc2a4	Mouse	Solute carrier family 2 member 4, GLUT4	Mm00436615_m1
Nppa	Mouse	Natriuretic peptide A	Mm01255747_g1
Nppb	Mouse	Natriuretic peptide B	Mm01255770_g1
Atp2a2	Mouse	ATPase sarcoplasmic/endoplasmic reticulum Ca^{2+} transporting 2	Mm01201431_m1
Myh6	Mouse	Myosin heavy chain 6	Mm00440359_m1
Myh7	Mouse	Myosin heavy chain 7	Mm00600555_m1
Tnnt2	Mouse	Troponin T2, cardiac type	Mm01290256_m1
Tnni3	Mouse	Troponin I3, cardiac type	Mm00437164_m1
Tpm1	Mouse	Tropomyosin 1	Mm00445895_g1
Actc1	Mouse	Actin alpha cardiac muscle 1	Mm01333821_m1
Ywhaz	Human	Tyrosine 3-monooxygenase/tryptophan 5-monooxygenase activation protein zeta	Hs01122445_g1

Gapdh	Human	Glyceraldehyde-3-phosphate dehydrogenase	Hs02786624_g1

*All assays were purchased from ThermoFisher Scientific.

Table S3 Demographic and clinical data from 5 LVAD patients

**Patient (symbol color used on Fig. S3)**	**Age at implant (years)**	**Gender**	**Race/Ethnicity**	**LVAD support duration (days)**	**Diagnosis**
Blue	67	Male	White	306	Idiopathic dilated cardiomyopathy
Red	21	Female	White	161	Idiopathic dilated cardiomyopathy
Green	53	Male	Asian	145	Idiopathic dilated cardiomyopathy
Orange	25	Male	Hispanic	501	Idiopathic dilated cardiomyopathy
Black	43	Male	White	67	Idiopathic dilated cardiomyopathy

Supplementary Figure legends

Fig. S1 Sustained induction of cardiac NR4A2 increases heart weight without affecting body mass. **a-b** Comparison of body weight (**a**) and heart weight normalized to body weight (**b**) between *Nr4a2*-icTg mice injected with tamoxifen (tam; n=26 males/18 females) or vehicle (n=29 males/19 females) and Cre recombinase expressing control mice (n=34 males/21 females) at 21 days following treatment. Data are expressed as mean±SEM. Data were analyzed by one-way ANOVA with Tukey test. * P<0.05 vs. Cre control+tam and † P<0.05 vs. *Nr4a2*-icTg+vehicle

Fig. S2 Cardiac myocyte-specific induction of NR4A2 induces left ventricular dilation. **a** Comparison of heart weight normalized to tibia length between *Nr4a2*-icTg mice injected with tamoxifen (tam; n=2 males/3 females) or vehicle (n=4 males/4 females) and Cre recombinase expressing control mice (n=4 males/4 females) at 28 days following treatment. **b-d** Echocardiographic assessment of LV posterior wall at end-diastole (LVPWd; **b**), LV internal diameter at end-diastole (LVIDd; **c**) and LV end-diastolic volume (LVEDV; **d**) in the same animals at 28 days following treatment. Data are expressed as mean±SEM. Data were analyzed by one-way ANOVA with Tukey test. * P<0.05 vs. Cre control+tam and † P<0.05 vs. *Nr4a2*-icTg+vehicle.

Fig. S3 NR4A2 expression tends to decrease in the left ventricle of heart failure patients following left ventricular assist device implantation. NR4A2 mRNA levels were quantified from 5 patients diagnosed with idiopathic cardiomyopathy (see Table S3 for individual demographic and clinical data) using left ventricular tissue collected at times of LVAD implantation (Imp) and explantation (Exp). Data were analyzed by paired Student *t*-test.

Fig. S4 Cardiac myocyte-specific induction of NR4A2 increases extracellular matrix turnover and left ventricular (LV) remodeling in the adult heart. **a** Top Reactome pathways linked to extracellular matrix remodeling, vesicle-mediated transport and other cellular functions that are significantly altered in the LV of *Nr4a2*-icTg at 21 days after tamoxifen injection when compared to the LV of Cre recombinase expressing control mice. Results generated from RNA-sequencing analysis of n=6 male mice per group. **b** Quantification by RNA-sequencing and real-time PCR of mRNAs encoding glucose transporters (*Slc2a1*, *Slc2a4*), markers of fetal gene reprogramming (*Nppa*, *Nppb*, *Atp2a2*, *Myh6*, *Myh7*) and cardiac sarcomeric proteins (*Tnnt2*, *Tnni3*, *Tpm1*, *Actc1*) in the left ventricle (LV) of *Nr4a2*-icTg mice at 21 days after tamoxifen (tam) treatment. Data are mean±SEM of n=6 animals per group and are expressed in fold change from expression levels detected in the LV of Cre recombinase expressing control mice. Data were analyzed by two-tailed Student *t*-test. * P<0.05 vs. Cre control+tam.

Fig. S5 Cardiac myocyte-specific induction of NR4A2 leads to up-regulation of glycolytic enzymes in the adult heart. Schematic of the glycolytic pathway accompanied by a comparison of mRNA expression levels for each enzyme of the pathway in the left ventricle of *Nr4a2*-icTg mice and Cre recombinase expressing control mice at 21 days after tamoxifen treatment. Enzymes expression levels were determined by RNA-sequencing and are expressed as mean±SEM fragments per kilobase of transcript per million mapped reads (FPKM) values from n=6 animals per group. Data were analyzed by two-tailed Student *t*-test. * P<0.05 vs. Cre control+tam.

Fig. S6 Acute induction of NR4A2 enhances the translational capacity of adult rat ventricular myocytes (ARVMs). ARVMs were transduced with adenoviral vectors to express human NR4A2 or enhanced green fluorescence protein (GFP) as control and RNA-sequencing was performed at 48 hours posttransduction. **a** Voronoi view of RNA-sequencing data revealing the main biological processes differentially regulated in ARVMs overexpressing NR4A2 when compared to GFP expressing ventricular myocytes. **b** The top Reactome pathways that are significantly altered in ARVMs overexpressing NR4A2 are all linked to protein translation and the post-translational modification of proteins. Data are based on analysis of 6 independent experiments using ARVMs isolated from different rats

Figure S1

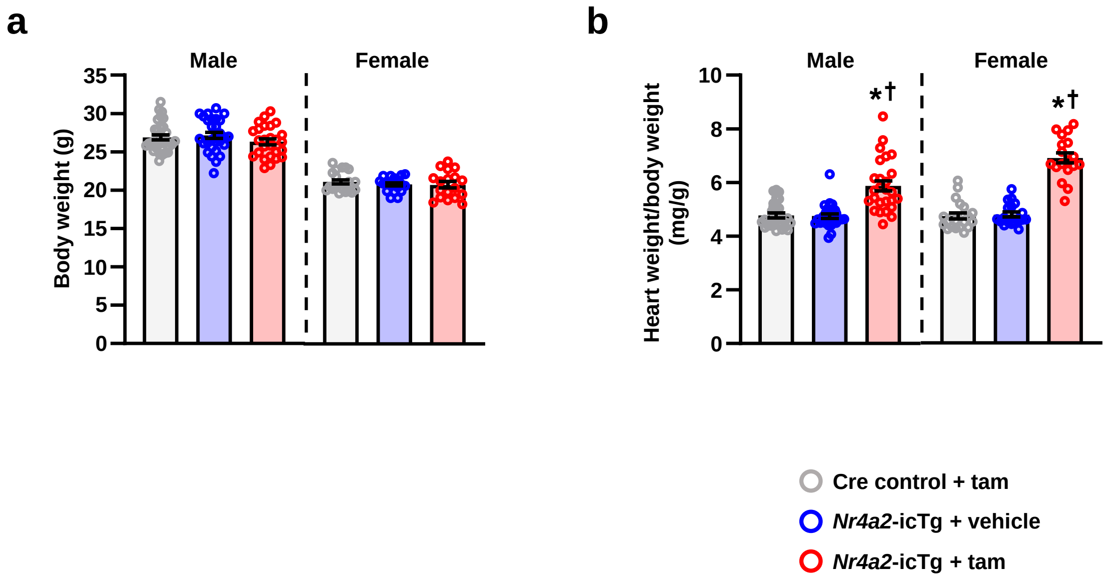
a
Male
Female
Body weight (g)
35
30
25
20
15
10
5
0
b
Male
Female
Heart weight/body weight (mg/g)
10
8
6
4
2
0
*†
*†
Cre control + tam
Nr4a2-icTg + vehicle
Nr4a2-icTg + tam

Figure S2

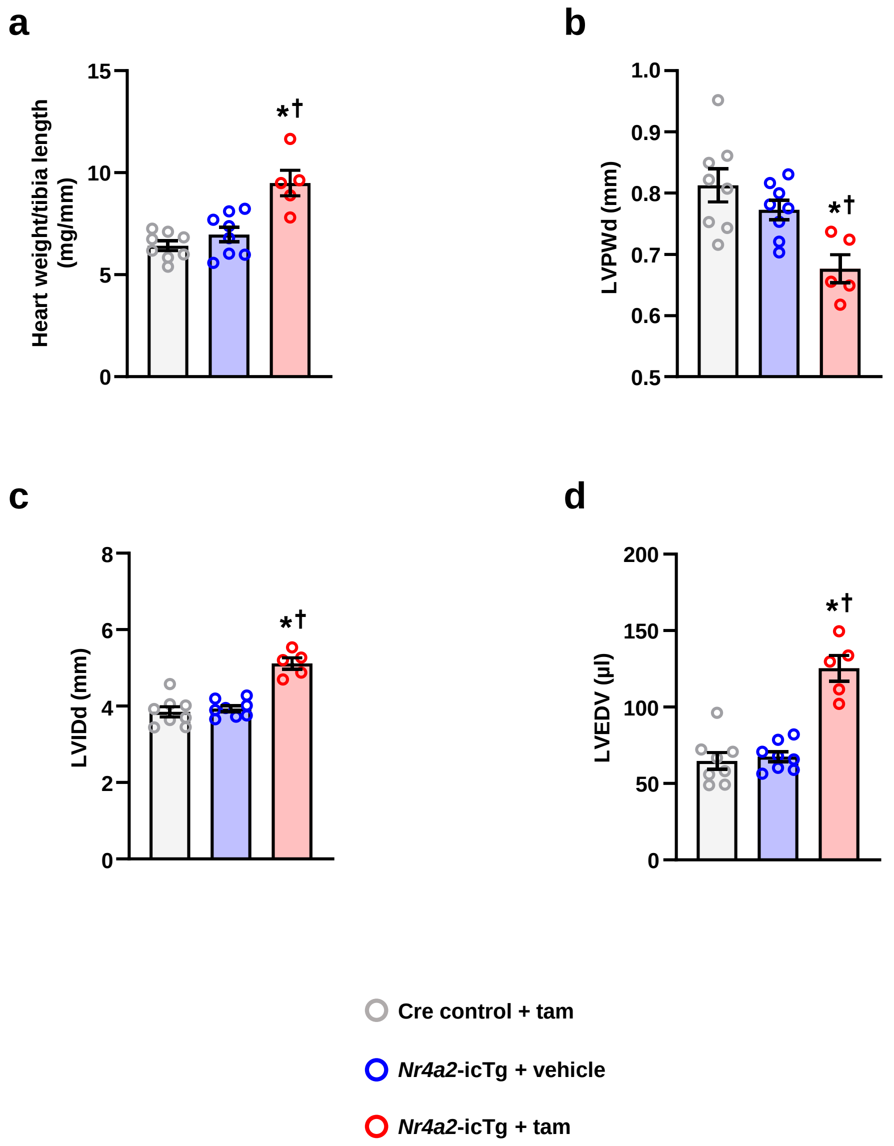
a
15
10
5
0
Heart weight/tibia length (mg/mm)
*†
b
1.0
0.9
0.8
0.7
0.6
0.5
LVPWd (mm)
*†
c
8
6
4
2
0
LVIDd (mm)
*†
d
200
150
100
50
0
LVEDV (µl)
*†
Cre control + tam
Nr4a2-icTg + vehicle
Nr4a2-icTg + tam

Figure S3

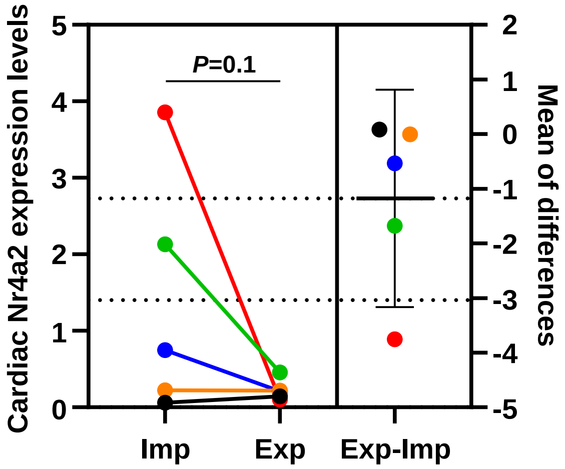

Cardiac Nr4a2 expression levels
Mean of differences
P=0.1
5
4
3
2
1
0
2
1
0
-1
-2
-3
-4
-5
Imp
Exp
Exp-Imp

Figure S4

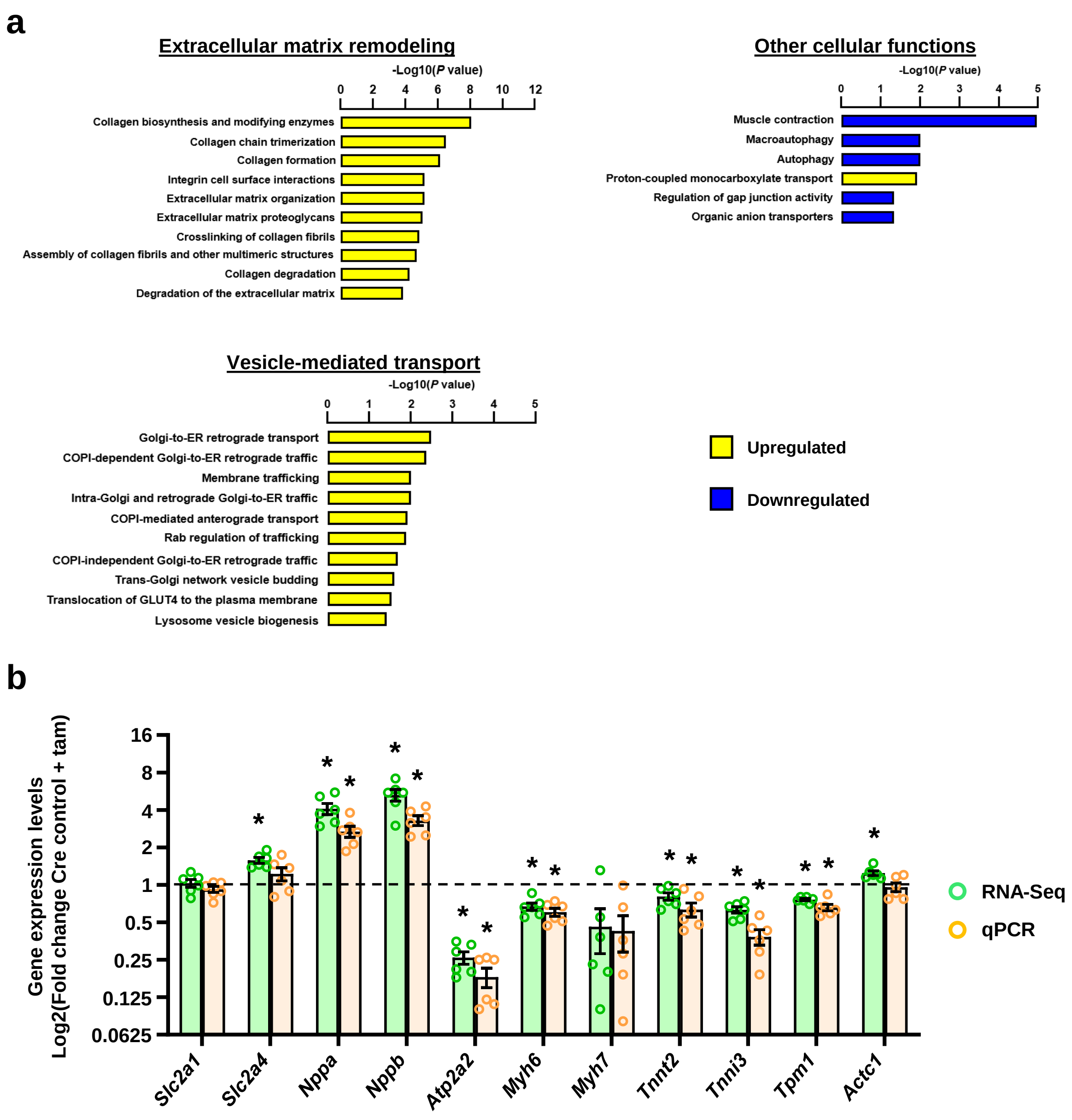
a
Extracellular matrix remodeling
-Log10(P value)
0 2 4 6 8 10 12
Collagen biosynthesis and modifying enzymes
Collagen chain trimerization
Collagen formation
Integrin cell surface interactions
Extracellular matrix organization
Extracellular matrix proteoglycans
Crosslinking of collagen fibrils
Assembly of collagen fibrils and other multimeric structures
Collagen degradation
Degradation of the extracellular matrix
Other cellular functions
-Log10(P value)
0 1 2 3 4 5
Muscle contraction
Macroautophagy
Autophagy
Proton-coupled monocarboxylate transport
Regulation of gap junction activity
Organic anion transporters
Vesicle-mediated transport
-Log10(P value)
0 1 2 3 4 5
Golgi-to-ER retrograde transport
COPI-dependent Golgi-to-ER retrograde traffic
Membrane trafficking
Intra-Golgi and retrograde Golgi-to-ER traffic
COPI-mediated anterograde transport
Rab regulation of trafficking
COPI-independent Golgi-to-ER retrograde traffic
Trans-Golgi network vesicle budding
Translocation of GLUT4 to the plasma membrane
Lysosome vesicle biogenesis
Upregulated
Downregulated
b
Gene expression levels
Log2(Fold change Cre control + tam)
16
8
4
2
1
0.5
0.25
0.125
0.0625
Slc2a1
Slc2a4
Nppa
Nppb
Atp2a2
Myh6
Myh7
Tnnt2
Tnni3
Tpm1
Actc1
RNA-Seq
qPCR

Figure S5

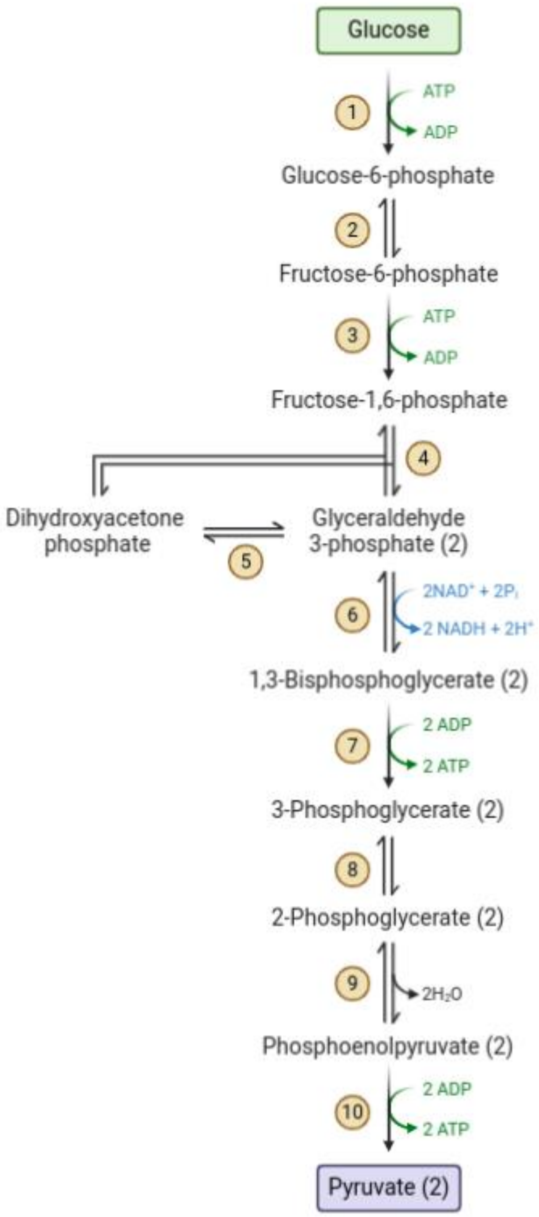

Enzyme	Gene Symbol	Cre Control FPKM	*Nr4a2*-icTg FPKM
1) Hexokinase	*Hk1*<br>*Hk2*	103±4<br>146±3	96±5<br>74±3*
2) Phosphoglucose isomerase	*Gpi1*	372±4	527±12*
3) Phosphofructokinase-1	*Pfkm*	819±8	1845±111*
4) Aldolase	*Aldoa*	3248±71	5549±193*
5) Triosephosphate isomerase	*Tpi1*	846±19	1548±82*
6) Glyceraldehyde 3-phosphate dehydrogenase	*Gapdh*	3018±103	5268±111*
7) Phosphoglycerate kinase	*Pgk1*	483±9	1655±155*
8) Phosphoglyceromutase	*Pgam1*<br>*Pgam2*	92±4<br>1133±31	175±20*<br>2353±99*
9) Enolase	*Eno1*<br>*Eno3*	102±6<br>1733±23	143±7*<br>8042±462*
10) Pyruvate kinase	*Pkm*	1043±24	2989±152*

Figure S6

a

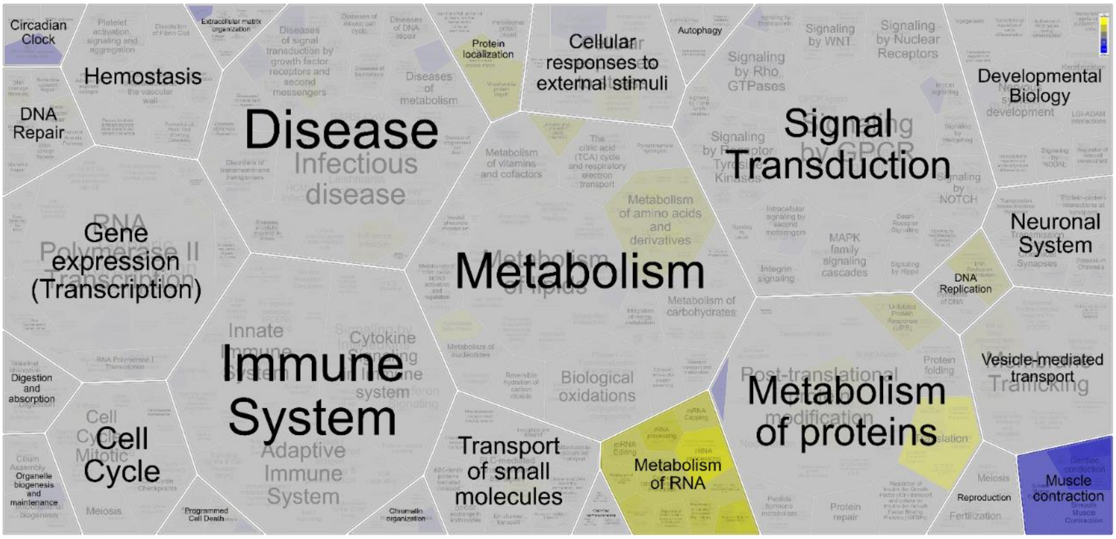
Circadian Clock
Hemostasis
DNA Repair
Disease
Infectious disease
Protein localization
Cellular responses to external stimuli
Autophagy
Signaling by WNT
Signaling by Nuclear Receptors
Signaling by Rho GTPases
Developmental Biology
Signal Transduction
Signaling by GPCR
Gene expression (Transcription)
Metabolism
Metabolism of amino acids and derivatives
Metabolism of lipids
Metabolism of carbohydrates
MAPK family signaling cascades
Integrin signaling
Neuronal System
DNA Replication
Innate Immune System
Cytokine Signaling in Immune system
Immune System
Adaptive Immune System
Digestion and absorption
Cell Cycle
Cell Cycle, Mitotic
Meiosis
Programmed Cell Death
Chromatin organization
Transport of small molecules
Biological oxidations
Metabolism of RNA
Post-translational protein modification
Metabolism of proteins
Vesicle-mediated transport
Membrane Trafficking
Protein folding
Protein repair
Reproduction
Fertilization
Muscle contraction
Organelle biogenesis and maintenance

b

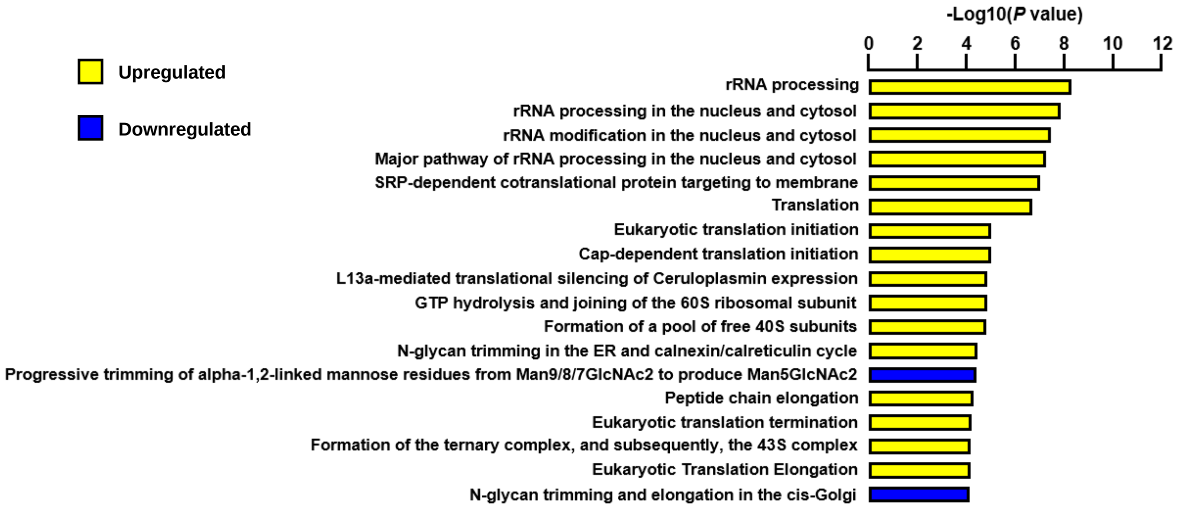
Upregulated
Downregulated
-Log10(P value)
0
2
4
6
8
10
12
rRNA processing
rRNA processing in the nucleus and cytosol
rRNA modification in the nucleus and cytosol
Major pathway of rRNA processing in the nucleus and cytosol
SRP-dependent cotranslational protein targeting to membrane
Translation
Eukaryotic translation initiation
Cap-dependent translation initiation
L13a-mediated translational silencing of Ceruloplasmin expression
GTP hydrolysis and joining of the 60S ribosomal subunit
Formation of a pool of free 40S subunits
N-glycan trimming in the ER and calnexin/calreticulin cycle
Progressive trimming of alpha-1,2-linked mannose residues from Man9/8/7GlcNAc2 to produce Man5GlcNAc2
Peptide chain elongation
Eukaryotic translation termination
Formation of the ternary complex, and subsequently, the 43S complex
Eukaryotic Translation Elongation
N-glycan trimming and elongation in the cis-Golgi
